# A Survival Metadata Analysis Responsive Tool (SMART) for web-based analysis of patient survival and risk

**DOI:** 10.1038/s41598-018-31290-z

**Published:** 2018-08-27

**Authors:** Yuan-Chia Chu, Wen-Tsung Kuo, Yuan-Ren Cheng, Chung-Yuan Lee, Cheng-Ying Shiau, Der-Cherng Tarng, Feipei Lai

**Affiliations:** 1 Graduate Institute of Biomedical Electronic & Bioinformatics, National Taiwan University, Taipei, 10617 Taiwan; 2 Graduate Institute of Computer Science & Information Engineering, National Taiwan University, Taipei, 10617 Taiwan; 3 Department of Life Science, National Taiwan University, Taipei, 10617 Taiwan; 4 Department of Radiology, School of Medicine, National Yang-Ming University, Taipei, 11221 Taiwan; 5 Institute of Biomedical Sciences, Academia Sinica, Taipei, 11574 Taiwan; 6 Division of Radiation Oncology, Department of Oncology, Taipei Veterans General Hospital, Taipei, 11217 Taiwan; 7 Information Management Office, Taipei Veterans General Hospital, Taipei, 11217 Taiwan; 8 Institute of Clinical Medicine, National Yang-Ming University, Taipei, 11221 Taiwan; 9 Department and Institute of Physiology, National Yang-Ming University, Taipei, 11221 Taiwan; 10 Division of Nephrology, Department of Medicine, Taipei Veterans General Hospital, Taipei, 11217 Taiwan

**Keywords:** Cancer, Risk factors

## Abstract

Health information systems contain extensive amounts of patient data. Information relevant to public health and individuals’ medical histories are both available. In clinical research, the prediction of patient survival rates and identification of prognosis factors are major challenges. To alleviate the difficulties related to these factors, Metadata Utilities was developed to help researchers manage column definitions and information such as import/query/generator Metadata files. These utilities also include an automatic update mechanism to ensure consistency between the data and parameters of the batch produced in the conversion procedure. Survival Metadata Analysis Responsive Tool (SMART) provides a comprehensive set of statistical tests that are easy to understand, including support for analyzing nominal variables, ordinal variables, interval variables or ratio variables as means, standard deviations, maximum values, minimum values, and percentages. In this article, the development of a raw data source and transfer mechanism, Extract-Transform-Load (ETL), is described for data cleansing, extraction, transformation and loading. We also built a handy method for data presentation, which can be customized to the trial design. As demonstrated here, SMART is useful for risk-adjusted baseline cohort and randomized controlled trials.

## Introduction

The cancer survival rate and progression-free survival rate are the most important measurements in cancer therapy research^1,2^. A survival analysis mainly uses a method designed to manage the dependent variable of time to an event. Survival analyses are extensively used in clinical and epidemiological follow-up studies^3–8^. In biomedical sciences, survival analyses are generally used for observations of the time to death of either patients or laboratory animals.

Commercial tools such as IBM SPSS Statistics, Stata^®^, and SAS^®^ are used in survival analyses to describe patient survival among demographic groups^9,10^. Survival analysis is a common analytical technique based on time to event analysis and requires a very complex analytical procedure. Recently, many applications have been developed to simplify this procedure, such as CanSurv^11^ and PODSE^12^. CanSurv is a statistical software tool designed to analyze population-based survival data. For grouped survival data, CanSurv can adapt to both the typical survival model and combination treatment survival models; it also provides various charts for modeling diagnoses. PODSE is an analytics optimizer for MATLAB. However, these platforms cannot be adapted to the many varieties of data structures in health information systems.

Survival Metadata Analysis Responsive Tool (SMART) is a web application based on the Shiny package in R. SMART is a public-domain software tool that utilizes a simple process to generate tables and figures that are appropriate for scientific publications. Two or more survival curves^13^ are commonly compared in survival analyses conducted in cohort and randomized controlled trials (RCTs). SMART is a multi-platform service that operates on Windows, Mac OS X, Linux/Unix, and mobile devices. This software may help to reduce researchers’ workloads.

SMART is freely available at http://140.112.30.202:3838/ for academic researchers to analyze their projects. The open-source code repository is available at https://goo.gl/ZH49SL. This study has two main objectives: to introduce the functions in SMART and to validate its survival analysis tools.

## Methods

### SMART web application

**Figure 1:**
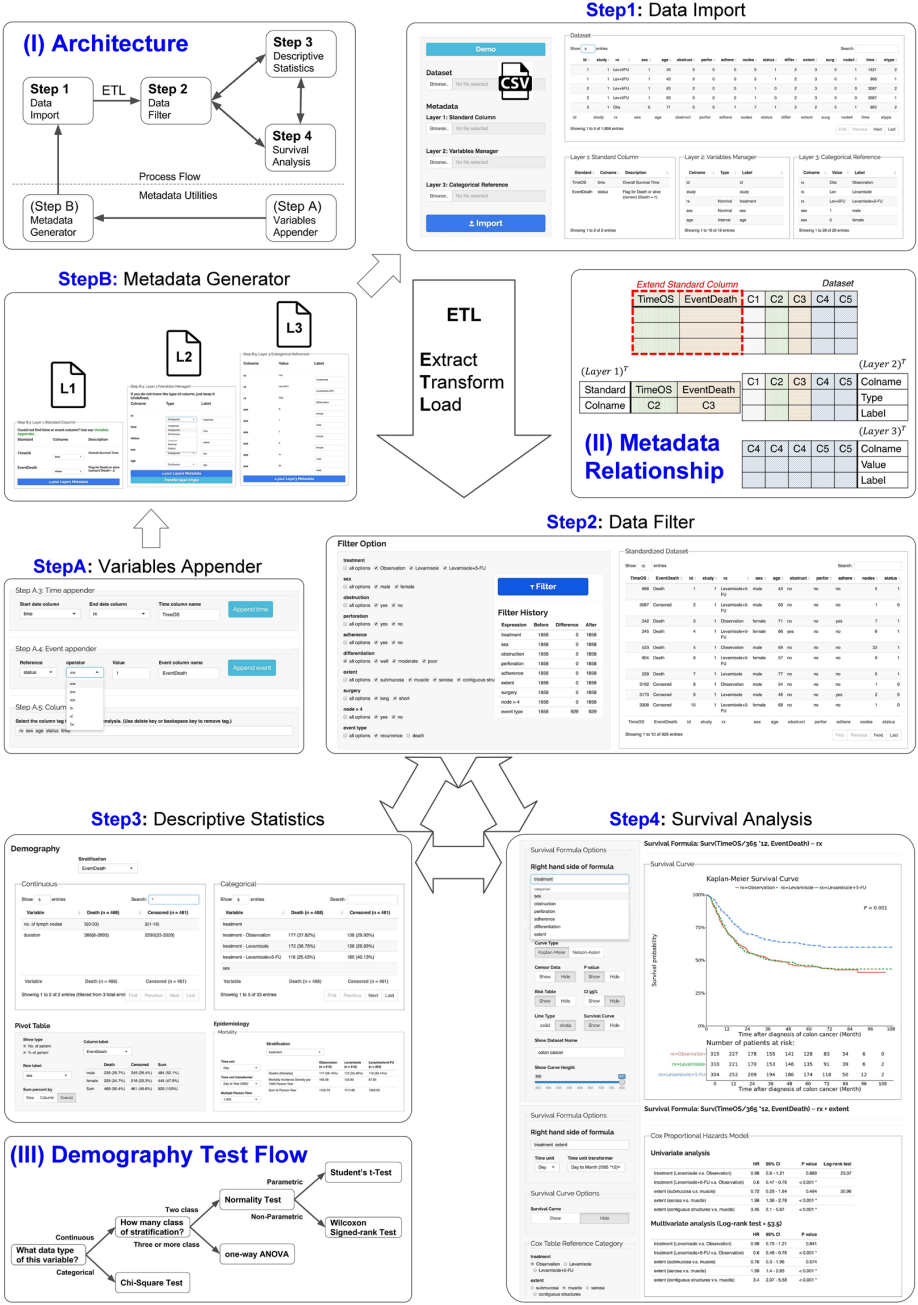
Workflow for SMART. Step A: The data management utilities of the survival analysis. Step B: The metadata management utilities of the survival analysis. Steps 1–4: SMART provides a uniform platform that comprises four analytical parts—data import (Step 1), data filtering (Step 2), statistical testing (Step 3), and a basic survival analysis and Cox proportional hazards regression analysis (Step 4).

SMART was developed to provide a standardized framework for the data obtained from survival analyses (Fig. 1) in cohort studies, RCTs, and time-to-event analyses. Researchers can easily create metadata files with Metadata Utilities (Fig. 1; Steps A and B). A simple comma-separated-values (CSV) file format is imported into SMART as the input. The Metadata Utilities are Extract-Transform-Load (ETL) tools^6,14^ that help users create a three-layer metadata data framework for further analysis.

The Metadata Generator (MG) is a supportive tool that transforms raw data into a standard data framework with various layers. Layer one is a standard column. The column contains dependent variable relationships that help describe the survival analysis. Variable relationships help researchers analyze the survival data and determine the most important variables. Thus, unwanted pedantic data formats are eliminated. Dependent variables are defined based on their raw data in this layer, such as the observed time (TimeOS) and death event (EventDeath).

**Figure 2:**
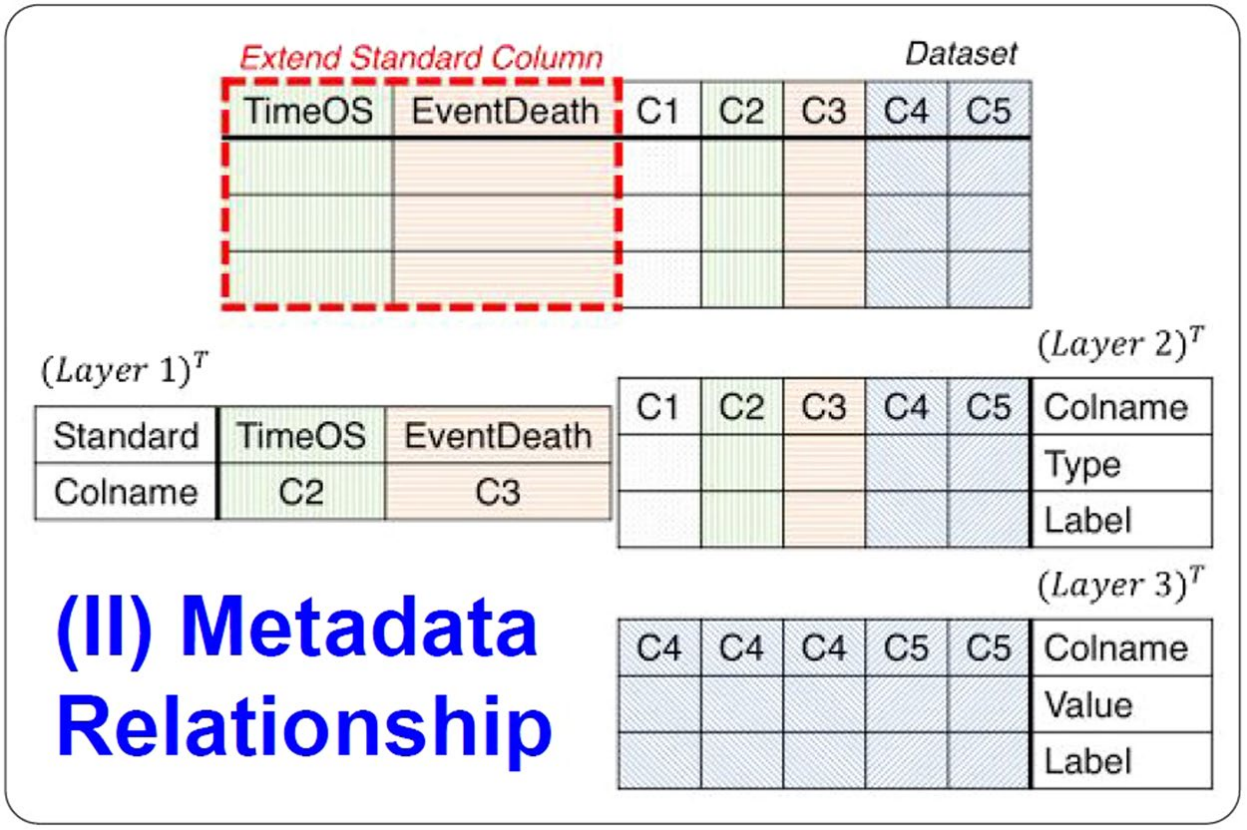
Metadata relationship in SMART. The “Extend Standard Column” is defined in layer 1, and the remaining data are defined in layer 2.

Layer two (Variable Manager) determines the control properties of the variables either automatically or manually. Layer three (Categorical Reference) creates a dictionary for categorical variables from the raw data. Users can also use this layer to map a numeric value as a factored variable. For example, the number one can represent “male” {1, m, M}, and the number zero can represent “female” {0, f, F}. Researchers can easily upload datasets and confirm their content. After uploading a dataset, users are prompted to complete a metadata template (Fig. 2).

### Metadata for data management and survival analysis

Metadata are descriptive data for a dataset and include information on how the data are stored or manipulated and on partial semantics, such as the implantation of the data. This information may comprise large changes that meet or exceed the variation in the data. Metadata are often considered an extension of the schema concept in structured databases. Typically, the metadata indicate constraints among the individual media objects, which are implicit and uncertain in the databases themselves. Some metadata captures information that does not depend on the data. Two of these types of metadata are the survival status and the time-to-event. The criterion we use to classify metadata^15^ is the degree to which they are successful in capturing the data and informative content from the information resource (called artifacts or documents in different contexts) present in various media types. The different types of metadata provide guidance for different queries or options for accessing information^16^.

In a survival analysis, researchers must specify a time-to-event variable, namely, a status variable, to determine the key point of the survival analysis. SMART must be used to handle raw data, time fields, and events. In SMART, we use three-layer metadata to accomplish these tasks. Layer one records the time and the event, layer two records the type of data in each column, and layer three records the meanings of the values. The next section introduces the relationships between the metadata (Fig. 2).

### Statistical testing for differences in the characteristics of the study subjects

**Figure 3:**
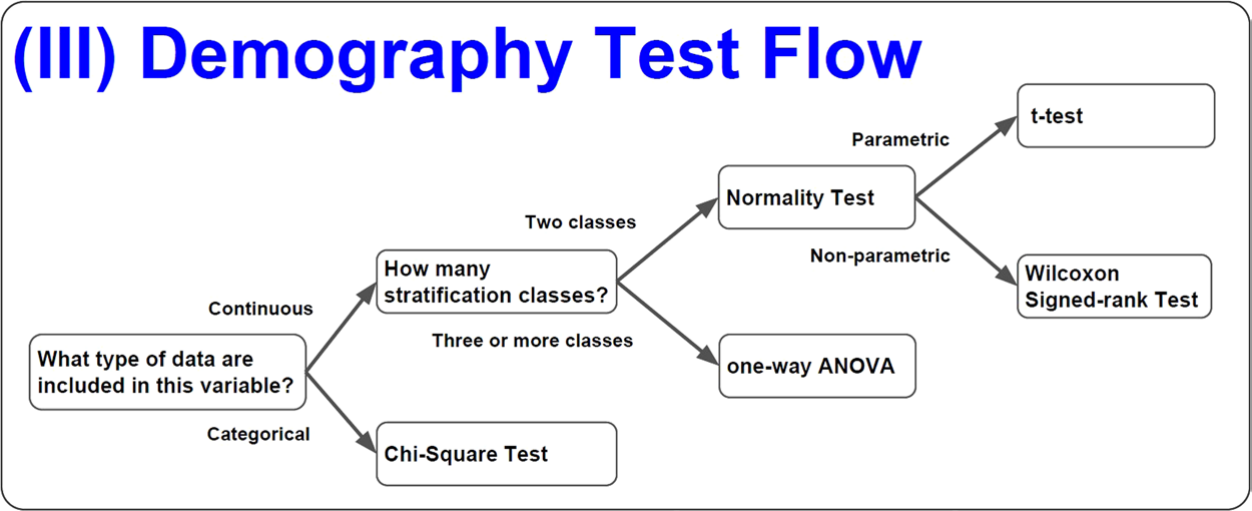
The demographic test flow in SMART. The demographic tests follow sequential test criteria for the data type, class of stratification, normality, and a defined test method.

A comprehensive comparison of the survival groups is important to determine the effects of cancer treatments between an experimental group and a control group. The baseline characteristics of the study subjects are effectively inspected with a mechanism that determines the proper model for the statistical analysis. SMART includes the Shapiro-Wilk (SW) test, the t-test, one-way analysis of variance (ANOVA), and other tests that are explained in the next paragraph (Fig. 3).

First, SMART assesses the normal distribution of all data within all independent numeric variables. We provide the SW test and Anderson-Darling (AD) test^17^ as methods for analyzing data normality. These tests are applicable to datasets of any size. Data within the independent variable will be categorized as either parametric or non-parametric data. Next, SMART uses t-tests for parametric statistical analyses of factors with more than two levels, whereas one-way ANOVA is used for statistical analysis for those trial designs containing more than two levels of the dependent variable, e.g., treatment, differentiation, extent(Fig. 1; Steps 3). For non-parametric variables, the Wilcoxon test is selected. The results obtained from numeric variables are displayed in continuous tables. For categorical variables, the chi-square distribution model for statistical analysis guarantees that the results will be displayed in the category part of the table.

### Survival analysis of differences in the variances in survival time

Generally, a survival analysis is a collection of statistical procedures for analyzing data in which the outcome variable of interest is the time to an event. This variable is typically the time to death or recurrence periods for cancer patients. In a time-to-death survival analysis, death is the event being analyzed, and the time is determined in an experimental setting. According to the 2015 Facility Oncology Registry Data Standard (FORDS)^18^, the initial diagnostic date can be recorded in any way that uses the first date of diagnosis, regardless of whether the data are based on clinical or histological parameters. The date of last contact or death will determine whether the patient was alive, was not contacted, or was deceased. Because censored data are generally used in survival analyses, the number of deceased people is estimated using a survival function $\hat{S}$ and a Kaplan-Meier (KM) survival curve to compare variances between two different types of survival data, or the log-rank test is used when the survival rate is constant over time. We denote *t*_(*n*)_ as a specific value for a random variable *T*, a time-to-event variable. $\hat{S}$ represents the survival probabilities, which are defined by the product of all fractions that estimate the conditional probabilities for failure times *t*_(*n*−1)_ and earlier time points. ${\hat{P}}_{r}$ represents conditional probabilities, with which we can estimate the survival probabilities for a specific time point. $\hat{S}({t}_{(n)})$ denotes the limit of probabilities for a conditional time point.

1 $$\hat{S}({t}_{(n)})=\hat{S}({t}_{(n-\mathrm{1)}})\times {\hat{P}}_{r}(T > {t}_{(n)}|T\ge {t}_{(n)})$$

Formula (1) represents the general KM formula. ($\hat{S}$ denotes survival probabilities)

2 $$\hat{S}({t}_{(n-\mathrm{1)}})=\prod _{i=1}^{n-1}{\hat{P}}_{r}(T > {t}_{(n)}|T\ge {t}_{(n)})$$

Formula (2) shows the KM formula = product limit of the probabilistic formula.

3 $$Log-rank\,statistic=\frac{{({O}_{2}-{E}_{2})}^{2}}{Var({O}_{2}-{E}_{2})}$$

Formula (3) represents the two-group case for the log-rank test formula.

The proportional hazards task performs a regression analysis of survival data based on the Cox proportional hazards (Coxph) model. This semi-parametric model is widely used to analyze survival data and explain the effects of explanatory variables on survival times. The statistical analysis factors used in Coxph models include the variables listed below. (a) The survival time specifies the variable to use as the survival time variable. The survival time is required. (b) The censoring variable specifies the variable that determines the censored cases. The censoring values are non-missing numeric values. The censoring variable is not required for a survival analysis. (c) Strata variables are stratified factors in the survival formula. SMART will first eliminate the missing values and fit the model based on levels of categorical factors in the survival formula.

If users do not specify the endpoint level for a numeric variable in the survival formula, SMART will determine the KM curve and Coxph based on a combination of unique values in the numeric variable. In formula 3, the summed observed score minus the expected score (*O*_*i*_ (observation value) − *E*_*i*_ (expected value)) is divided by the estimated variance to evaluate the p value based on a chi-squared distribution. In formula 4, *HR* denotes the hazard ratio, which is defined as the hazard for one individual divided by the hazard for another individual, and *β* denotes the exposure coefficient.

4 $$log(HR)=log(\frac{h(t)}{{h}_{0}(t)})={\beta }_{1}{X}_{1}+{\beta }_{2}{X}_{2}+\mathrm{...}+{\beta }_{p}{X}_{p}$$

Formula (4) represents the Coxph model.

### Demonstrative datasets

The dataset used in the demo mode of SMART is a public dataset from the RDatasets repository^19^. RDatasets is a public repository of 1039 datasets that were originally distributed with the statistical software environment R and some of its add-on packages. We adopted the colon data^10^ from the survival repository.

## Results

### SMART system

**Figure 4:**
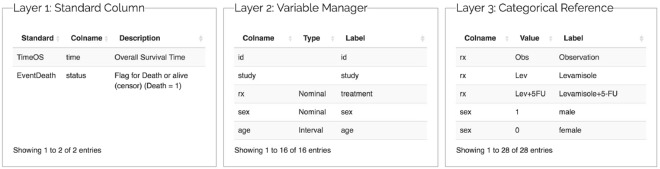
Metadata from the example dataset.

SMART comprises four steps to guide researchers in completing a new survival analysis. It helps researchers deal with the complex data from the Healthcare Information System (HIS). A specialized data framework is provided to help researchers find the dependent variables in the survival analysis, define the types of data representing dependent experimental variables, and abstract the data of the patient population. In the example dataset, TimeOS is the overall survival time, and EventDeath is a flag for “dead” or “alive” (“alive” = 0, “dead” = 1). The data censoring process is a complex procedure in the survival analysis. Two types of censoring are performed, including right and left censoring. The censored data represent the participation status of a subject during the study period. A right-censored subject refers to an event not included in the study period. In SMART, the right-censored method is majorly used for performing the survival analysis. The data framework in Step 1 of SMART is presented in Fig. 4.

The proposed survival analysis is to validate the efficacy of a therapy. SMART performs a KM survival analysis, the log-rank test, and the Coxph analysis in one click. Since SMART is a responsive web application, researchers do not need to rearrange the data to form a new study design. SMART is a perfect tool for iteratively testing hypotheses in cancer research and distinguishing true clinical contributions.

### The standard operating procedure for survival analyses in cancer research

**Figure 5:**
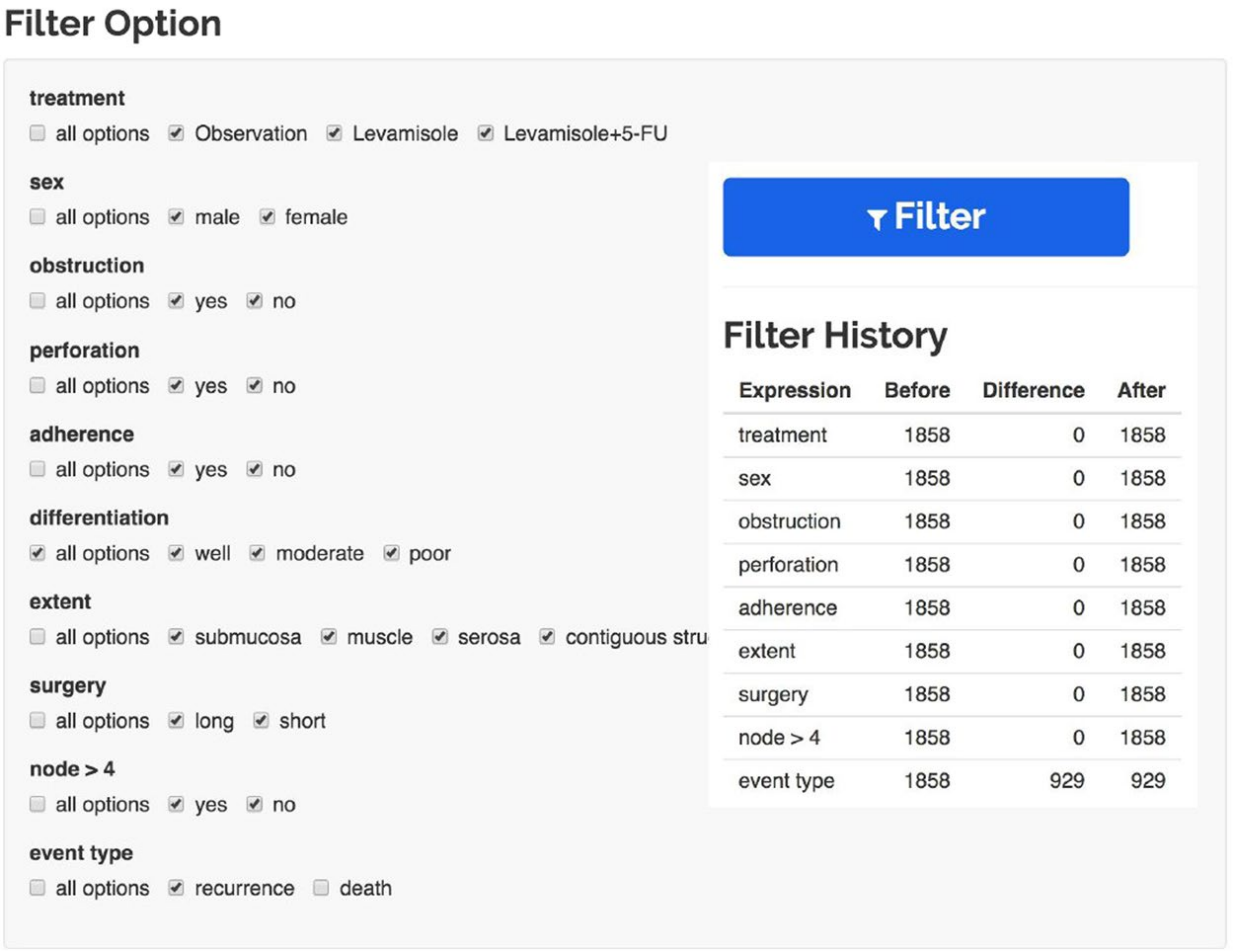
The experimental design. In this section, researchers can define their own inclusion and exclusion criteria using the filter and obtain the appropriate data based on the defined criteria.

We summarized the standard operating procedure for cancer research survival analyses based on the literature, and we automated the procedure on the SMART platform. SMART analyzes the data and identifies the dependent variables in Step 1. In Step 2, SMART helps researchers organize their experiments and define the inclusion and exclusion criteria. SMART can automatically locate missing data. In the example dataset, the excluded results could be easily identified when patients who underwent long surgical procedures were excluded from the study (Fig. 5).

**Figure 6:**
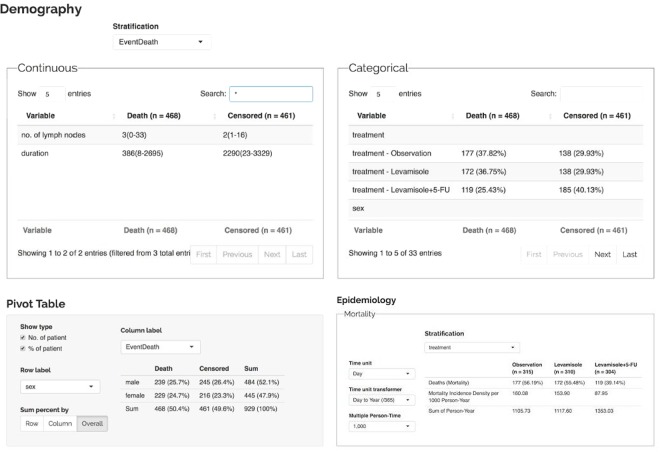
Data obtained from SMART. In this section, SMART shows the data by dividing variables into continuous and categorical data.

In Step 3, the data are abstracted to form demographic tables, in which we rearrange the results using various methods to validate the study design. Validation is performed because the likelihood of a mismatch or error occurring due to a conformational change is substantial if the design is flawed. In our example dataset (Fig. 6), we did not observe significant differences in age between deceased patients and living patients, but the number of affected lymph nodes was significantly higher in the deceased group than in the living group. A significantly lower (*P* < 0.001) mortality rate was also observed in the treatment group compared to the control group. We also applied the pivoting approach to the results and analyzed the epidemiology for our experimental design in Step 2.

In Step 4, we performed a KM survival analysis and the log-rank test to determine the outcome. In addition, SMART also identifies prognostic factors in the study using a Coxph analysis. The survival analysis implemented in SMART helps researchers and physicians analyze the survival rate in a defined period and the progression-free survival rate throughout the duration of therapy.

**Figure 7:**
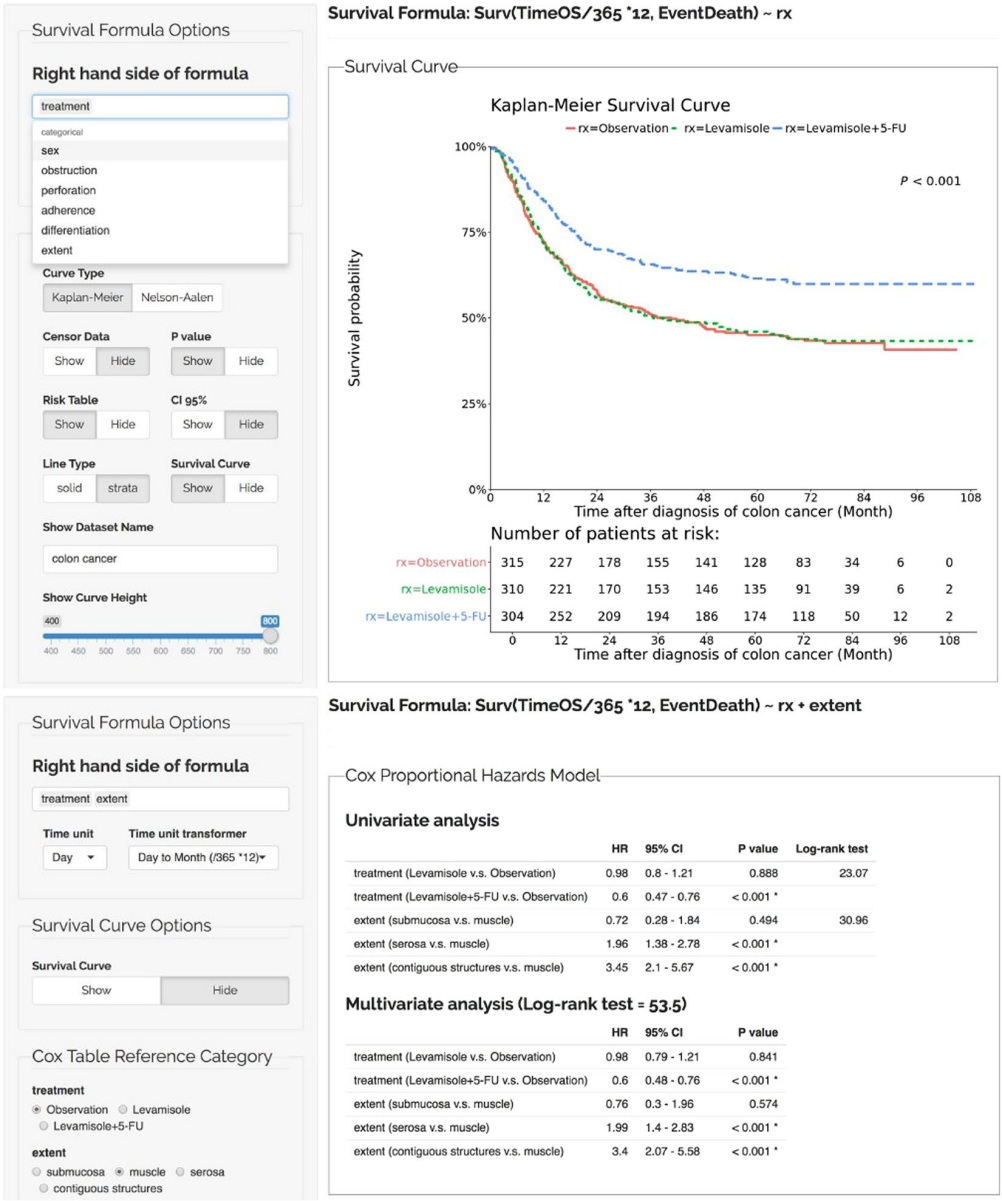
Representative results obtained from a stratified experimental design.

**Figure 8:**
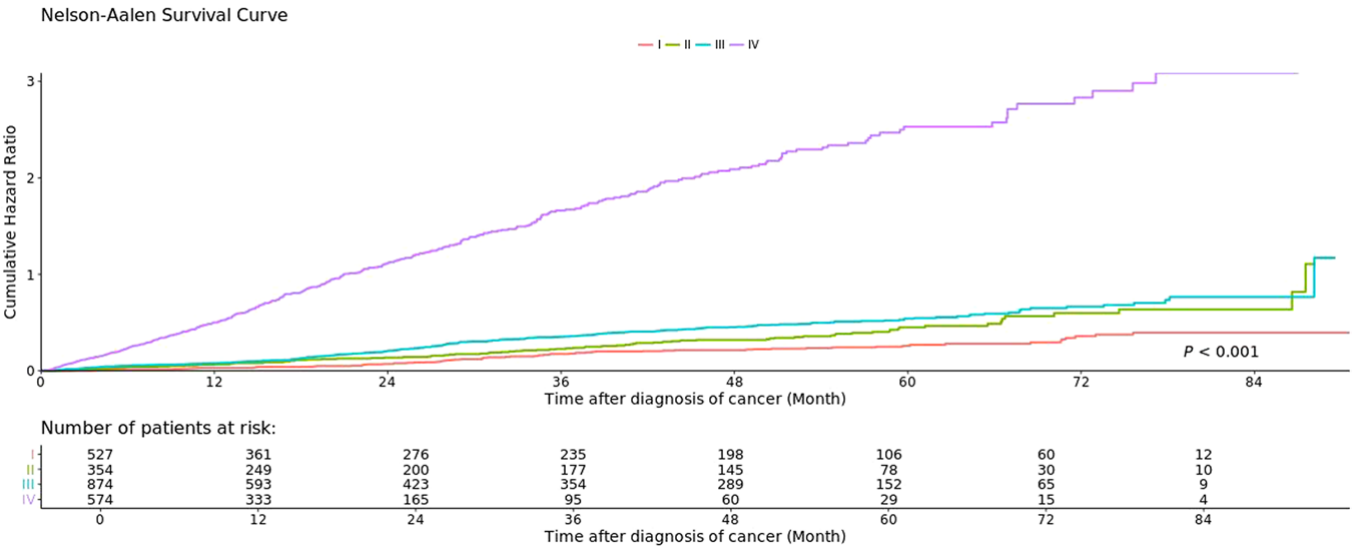
A real case of overall survival analysis by SMART.

In SMART, we can easily modify the analytical target and perform a stratified analysis by reformulating the survival formula in a few clicks. In our example dataset, we further stratified the treatment response according to the sex of the patient but did not observe a significant difference in survival rates between groups (Fig. 7). A clinical researcher was also requested to use SMART in the study, and SMART worked exactly as they desired. The result is adapted as a demonstration in the last figure (Fig. 8).

## Discussion

In clinical trials, two major issues must be discussed: the incidence of the event of interest and the time to the event in a study cohort. However, clinical researchers are usually frustrated by an inability to examine the real condition of patients. For instance, a cancer patient may die after the research period, and inclusion of these patients in the death event data is controversial. In addition, patients may transfer or be lost to follow up within the study period. These features lead to an uncertainty in event occurrence. The second important issue is the duration of time to the event occurrence. If the patient died within one month compared to six months, the medical intervention had completely different effects. However, the retrieval of the duration data from a HIS is always difficult^6^.

Survival analysis has been applied to clinical research to resolve these two major issues. This analysis involves an analysis of time-to event data designed to trace the patient’s outcome until the occurrence of an event, loss to follow up, or the end of the study. Therefore, the survival analysis not only assesses the death event but also examines recurrences, progression of disease, and the complete pathological response.

Web-based tools such as OASIS and SurvCurv have been widely implemented (refs^11,20,21^) and studies have demonstrated the use of various statistical analyses to identify potential research patient cohorts. There are several core concepts to this research, including access to open clinical data for research purposes, the design of a flexible research Metadata Utilities solution, and clarification of the characteristics of each study population. However, previous studies (refs^11,20–22^) did not propose how to integrate these solutions to a standard operation procedure process in the building of a cohort discovery. In the SMART, all of the solutions are combined into a standard operation procedure of survival analysis to accelerate the survey process of clinical research and decision support in the daily practice of clinical physicians. The most importantly, a high reusability infrastructure is provided for longitudinal clinical data.

In this article, the SMART system is demonstrated to assist clinicians in their studies^23^. This system supports a variety of measurement scales, discrete data, nominal data, ordinal data, or continuous interval data, depending on the complexity of the data types, and SMART can automatically map itself to the appropriate analysis method^23^. The process of justifying a prompt statistical model is time consuming, leading to a heavy workload for researchers. Therefore, the parser function in r is adopted to analyze the metadata and summarize the measurements of data to automatically normalize the study design for the selected model. The automated process greatly reduces the difficulty in choosing an appropriate study design and saves time.

Data presented in illustrations are crucial for clinical research. For instance, a diagram presents correlations and dependencies between the data and factors. A bar graph or a box chart is sufficient for comparisons of the differences between two groups or among three or more groups. A table provides comprehensive information, including study populations, ratios, and the statistical power of the results. However, these figures rely on various statistical models, and mastery of all statistical analyses requires time. In SMART, we provide an automated modeling tool and an interactive user interface. We reduced the effort required to perform data cleaning or solve issues of missing data in statistical models, which enabled clinicians to complete their research in an easy way. In our SMART system, we provided a user interface that helps researchers automatically preview the dataset, metadata configurations, descriptive statistics, and incidence density of the event, and draw the survival curve. Researchers can also adjust the data filter to generate a new study design and quickly obtain the results. They can modify the study dataset to exclude confounding factors while obtaining more precise results from the descriptive statistics.

In summary, we provide a web-based interactive survival analysis tool for clinicians. It is a simple and comprehensive tool for researchers in different medical fields. In the near future, we hope to connect SMART with the research database in hospitals and specially designed datasets from different divisions and improve the models using machine learning.

## Conclusions

SMART will assist researchers in handling their research data and testing hypotheses. Clinical researchers will be able to use SMART to more efficiently determine the overall survival rate, disease-free survival rate, and the Coxph from factors of interest. SMART will help researchers abstract their heterogeneous data without any command-line syntax or data reformation. It is also a free online software enabling users to perform their research.

We provide a standard operating procedure for survival analysis, including data ETL, data filtering, descriptive statistics, and survival analyses. SMART generates publication-ready tables and figures that are comparable with those derived from conventional statistical software. Furthermore, researchers can share their studies by sharing a token, ensuring that they can freely review and modify the analysis. This feature will stimulate collaboration and help projects succeed in an efficient way.

## Electronic supplementary material

SMART Supplementary

3. Dataset.csv

4. Layer1.csv

5. Layer2.csv

6. Layer3.csv
