# Author Correction: A Survival Metadata Analysis Responsive Tool (SMART) for web-based analysis of patient survival and risk

**DOI:** 10.1038/s41598-019-43446-6

**Published:** 2019-06-04

**Authors:** Yuan-Chia Chu, Wen-Tsung Kuo, Yuan-Ren Cheng, Chung-Yuan Lee, Cheng-Ying Shiau, Der-Cherng Tarng, Feipei Lai

**Affiliations:** 1 Graduate Institute of Biomedical Electronic & Bioinformatics, National Taiwan University, Taipei, 10617 Taiwan; 2 Graduate Institute of Computer Science & Information Engineering, National Taiwan University, Taipei, 10617 Taiwan; 3 Department of Life Science, National Taiwan University, Taipei, 10617 Taiwan; 4 Department of Radiology, School of Medicine, National Yang-Ming University, Taipei, 11221 Taiwan; 5 Institute of Biomedical Sciences, Academia Sinica, Taipei, 11574 Taiwan; 6 Division of Radiation Oncology, Department of Oncology, Taipei Veterans General Hospital, Taipei, 11217 Taiwan; 7 Information Management Office, Taipei Veterans General Hospital, Taipei, 11217 Taiwan; 8 Institute of Clinical Medicine, National Yang-Ming University, Taipei, 11221 Taiwan; 9 Department and Institute of Physiology, National Yang-Ming University, Taipei, 11221 Taiwan; 10 Division of Nephrology, Department of Medicine, Taipei Veterans General Hospital, Taipei, 11217 Taiwan

Correction to: *Scientific Reports* 10.1038/s41598-018-31290-z, published online 27 August 2018

In the original version of this Article, there were errors in Affiliations 6, which was incorrectly listed as ‘Division of Radiation Oncology, Cancer Center, Taipei Veterans General Hospital, Taipei, 11217, Taiwan’.

The correct affiliation is as follows:

‘Division of Radiation Oncology, Department of Oncology, Taipei Veterans General Hospital, Taipei, 11217, Taiwan’

This error has now been corrected in the PDF and HTML versions of the Article.

