# Supplementary material for: A Survival Metadata Analysis Responsive Tool (SMART) for web-based analysis of patient survival and risk
Source: Sci Rep. 2018 Aug 27;8:12880. doi: 10.1038/s41598-018-31290-z (PMC6110739; doi:10.1038/s41598-018-31290-z)

A Survival Metadata Analysis Responsive Tool (SMART) for web-based analysis of patient survival and risk

Demo website: http://140.112.30.202:3838

Yuan-Chia Chu, Wen-Tsung Kuo,
Yuan-Ren Cheng, Chung-Yuan Lee,
Cheng-Ying Shiau, Der-Cherng Tarng, and Feipei Lai

2018.07.30

Clinical Study Design

- Experimental Studies
  - **Randomised Controlled Trail (RCT)**
  - Non-Randomised Controlled Trail

- Observational Studies
  - Cohort Study
    - Prospective Cohort Study
    - **Retrospective Cohort Study**
  - Case-Control Study
  - Cross-Sectional Study

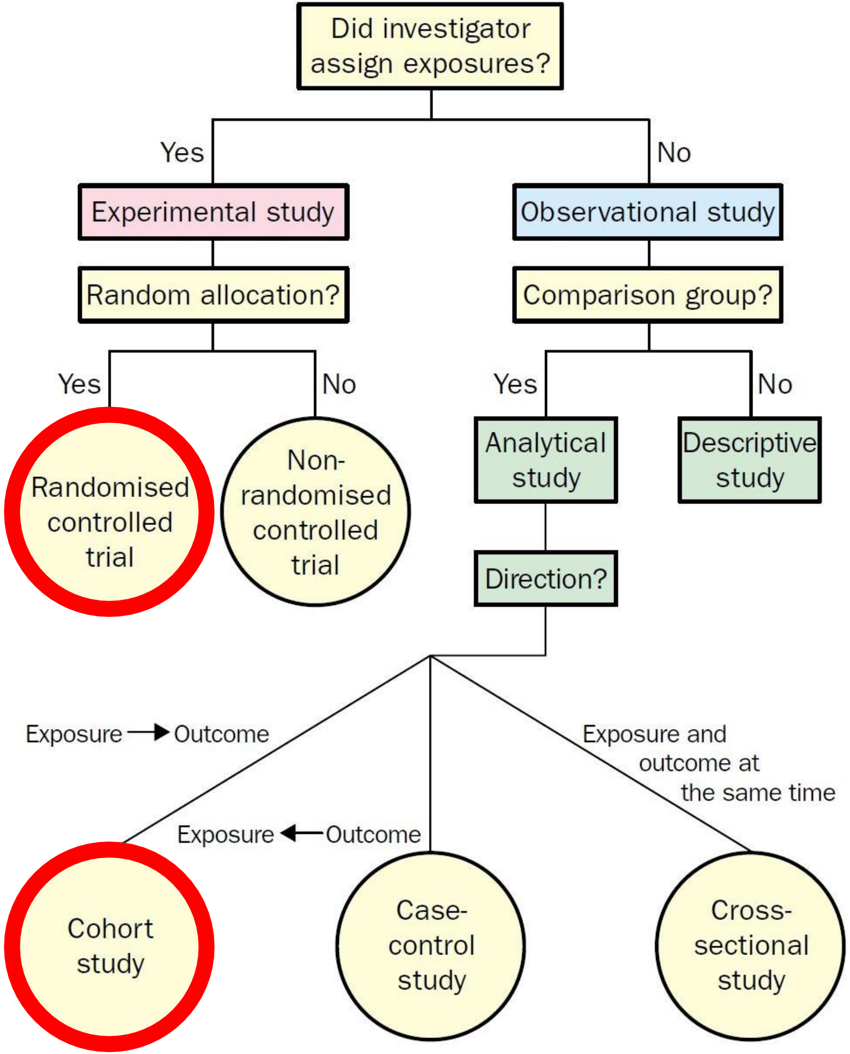

Ref: Grimes, David A., and Kenneth F. Schulz. "An overview of clinical research: the lay of the land." *The lancet* 359.9300 (2002): 57-61.

Epidemiologic Study Design

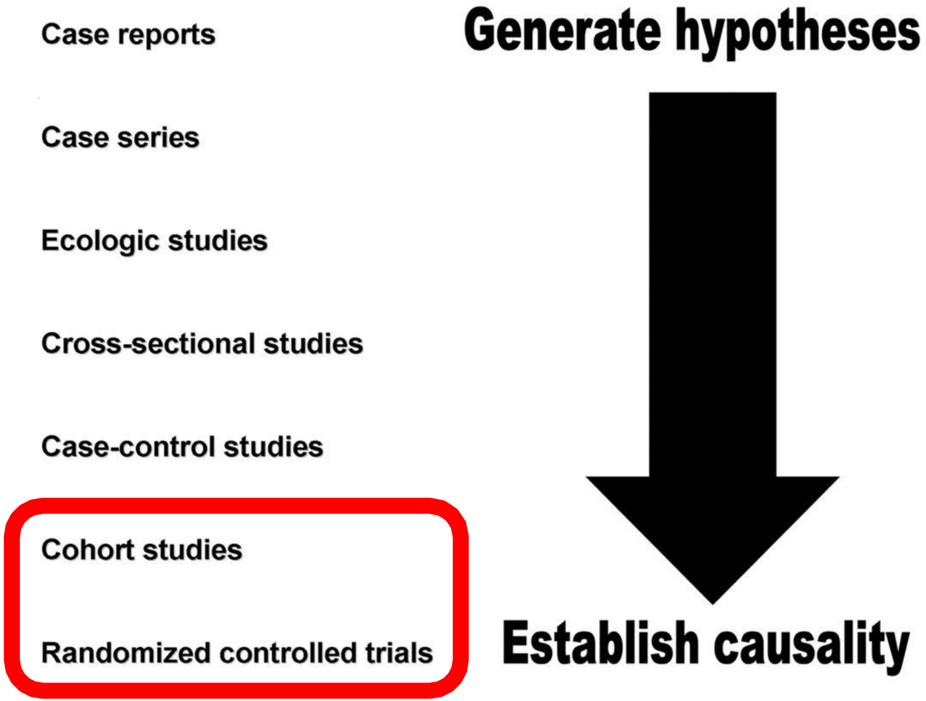

Ref: Tower, Richard L., and Logan G. Spector. "The epidemiology of childhood leukemia with a focus on birth weight and diet." *Critical reviews in clinical laboratory sciences* 44.3 (2007): 203-242.

Background

- **Importance**
  - massively used in clinical and epidemiological study
  - Cancer therapy research
- Recent Statistical Tools for Survival Analysis
  - SAS®
  - Stata®
  - SPSS
  - R

Commercial Utilities

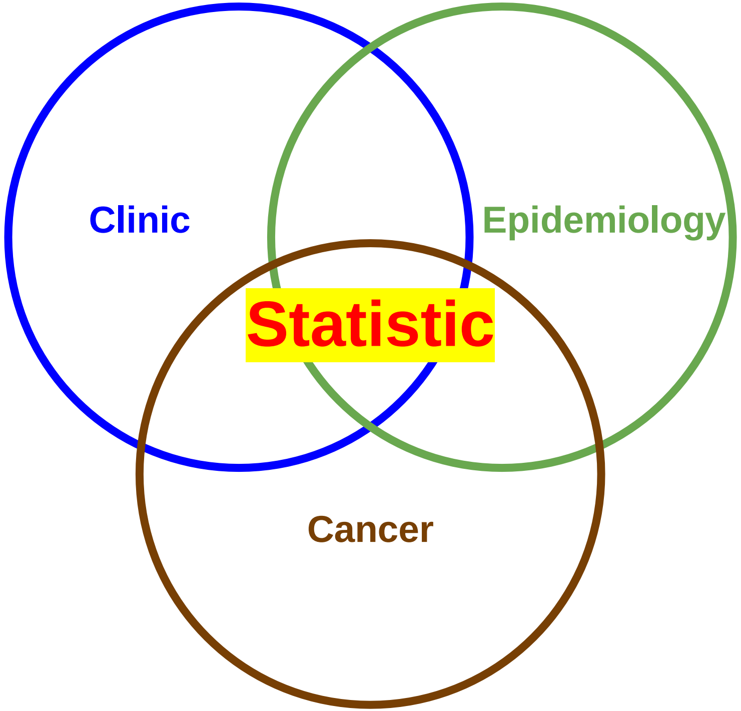

Motivation

- **Bottlenecks** in commercial utilities
  - **High cost** authorization
  - **Learning threshold** is high
  - **Time-consuming** when using it
  - **Many contraints** like input format
  - Impossible to collaborate

Objective

- Accessibility
- Learning Threshold
- Efficiency
- Collaboration
- Interactive
- Standardization

How to Achieve the Objective?

- **Survival Analysis**
- **Metadata**
- **Responsive Tool**

- **S**urvival **M**etadata **A**nalysis **R**esponsive **T**ool

Survival Analysis - Basic Concept

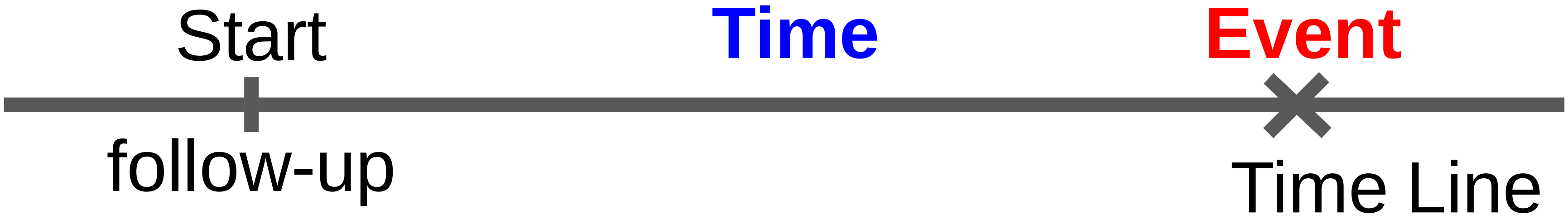

- Clinical time to event-based analysis
  - **Time** = follow-up time = overall survival time
  - **Event** = death or recurrence = failure

Survival Analysis - Censored Data

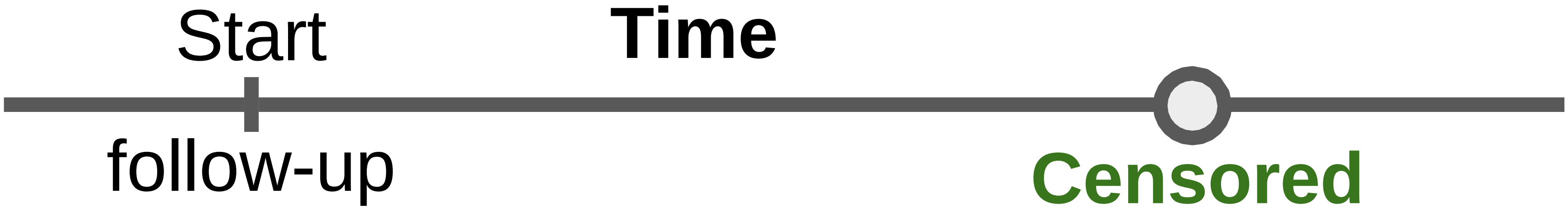

- Censored Data
  - No event occurs
  - Study end
  - Lost follow-up
- Data presentation
  - **1** = **event** occurs
  - **0** = **censored** data

Survival Analysis - Find Solution

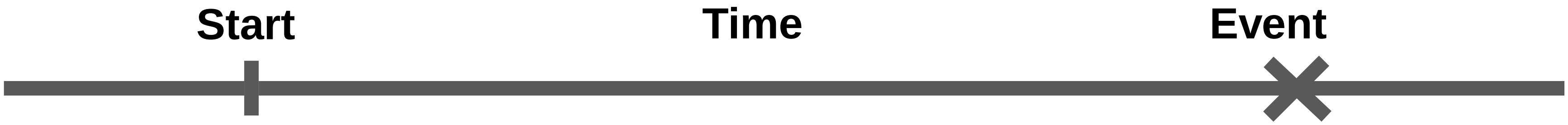

Which treatment is better?
Which are risk factors?

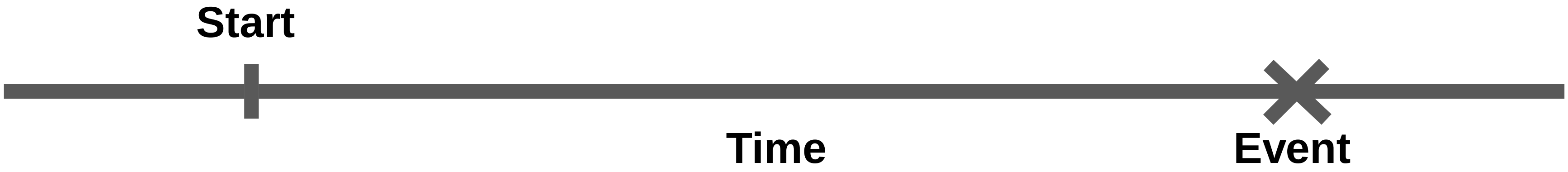

The Keypoint of Survival Analysis

- What is Time? What is Event?
- What is the data type of each column?
- What is the meaning of value?

X	trt	celltype	time	status	karno	diagtime	age	prior
1	1	squamous	72	1	60	7	69	0
2	1	squamous	411	1	70	5	64	10
3	1	squamous	[illegible]	1	[illegible]	3	38	0
4	1	squamous	126	1	60	9	63	10
5	1	squamous	118	1	70	11	65	10

Dataset

One Method of Describe Dataset

Time

Event

Treatment

Prior Therapy

X	trt	celltype	time	status	karno	diagtime	age	prior
1	1	squamous	72	1	60	7	69	0
2	1	squamous	411	1	70	5	64	10
3	1	squamous	[illegible]	1	[illegible]	3	38	0
4	1	squamous	126	1	60	9	63	10
5	1	squamous	118	1	70	11	65	10

Dataset

Yes

No

Smart Method of Describe Dataset

Metadata

Standard	Colname
TimeOS	time
EventDeath	status

X	trt	celltype	time	status	karno	diagtime	age	prior
ID	Treatment	Cell Type	Duration	Event	Karnofsky score	Diagnosis to randomisation in month	Age	Prior therapy
	Categorical	Categorical			Continuous	Continuous	Continuous	Categorical

Colname	Value	Label
trt	1	standard
trt	2	test
prior	10	yes
prior	0	no

Dataset

X	trt	celltype	time	status	karno	diagtime	age	prior
1	1	squamous	72	1	60	7	69	0
2	1	squamous	411	1	70	5	64	10
3	1	squamous	[illegible]	[illegible]	[illegible]	3	38	0
4	1	squamous	126	1	60	9	63	10
5	1	squamous	118	1	70	11	65	10

Metadata Relationship

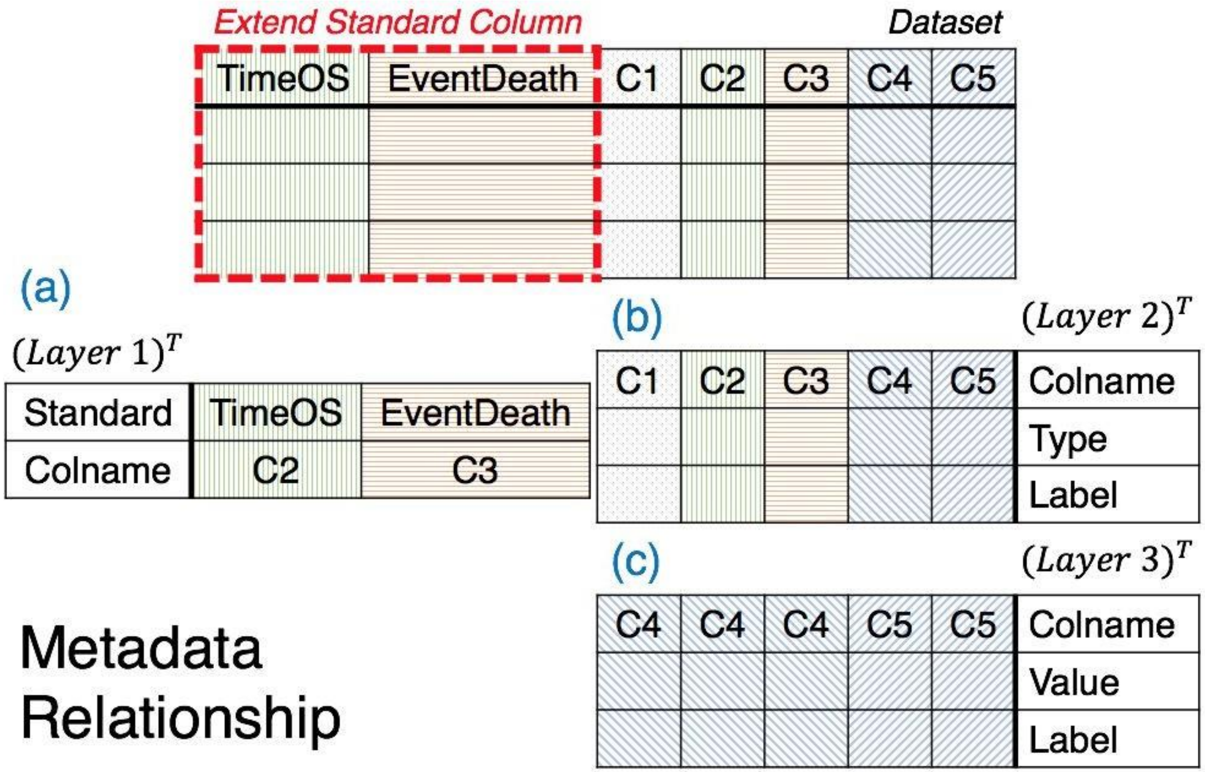

Layer 1 Metadata

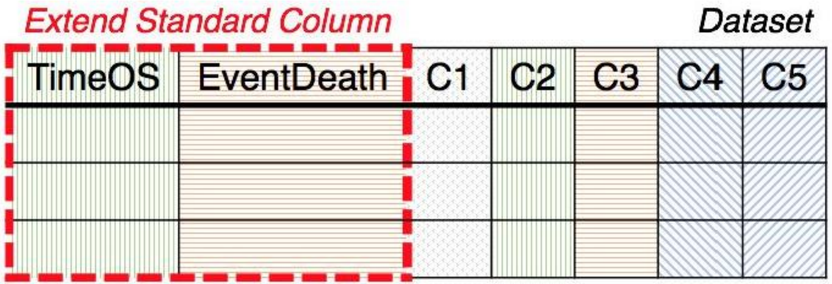

(a)

$(Layer\ 1)^T$

Standard	TimeOS	EventDeath
Colname	C2	C3

Layer 2 Metadata

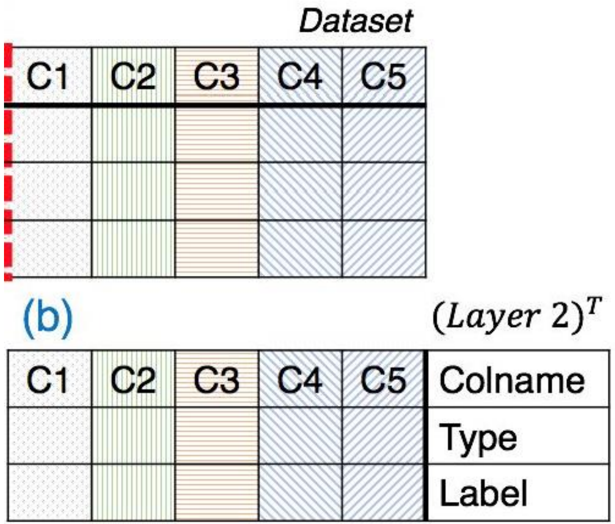

Layer 3 Metadata

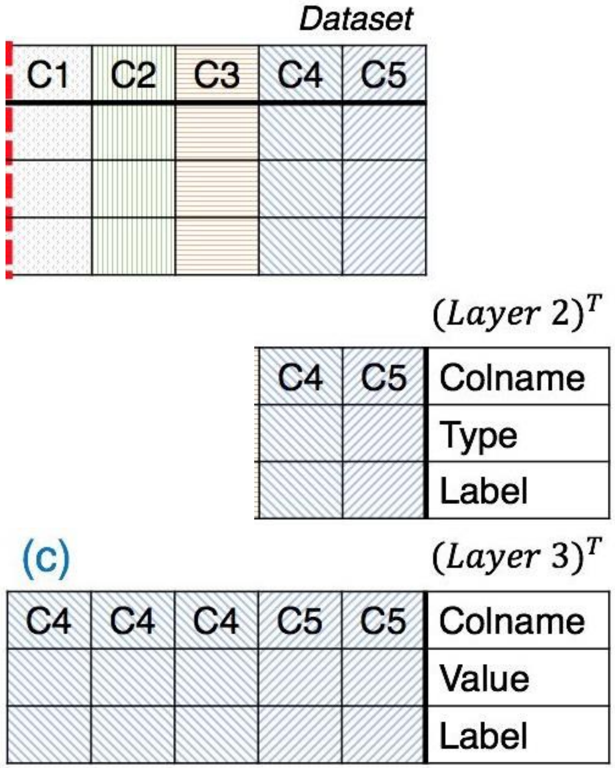

Metadata Relationship

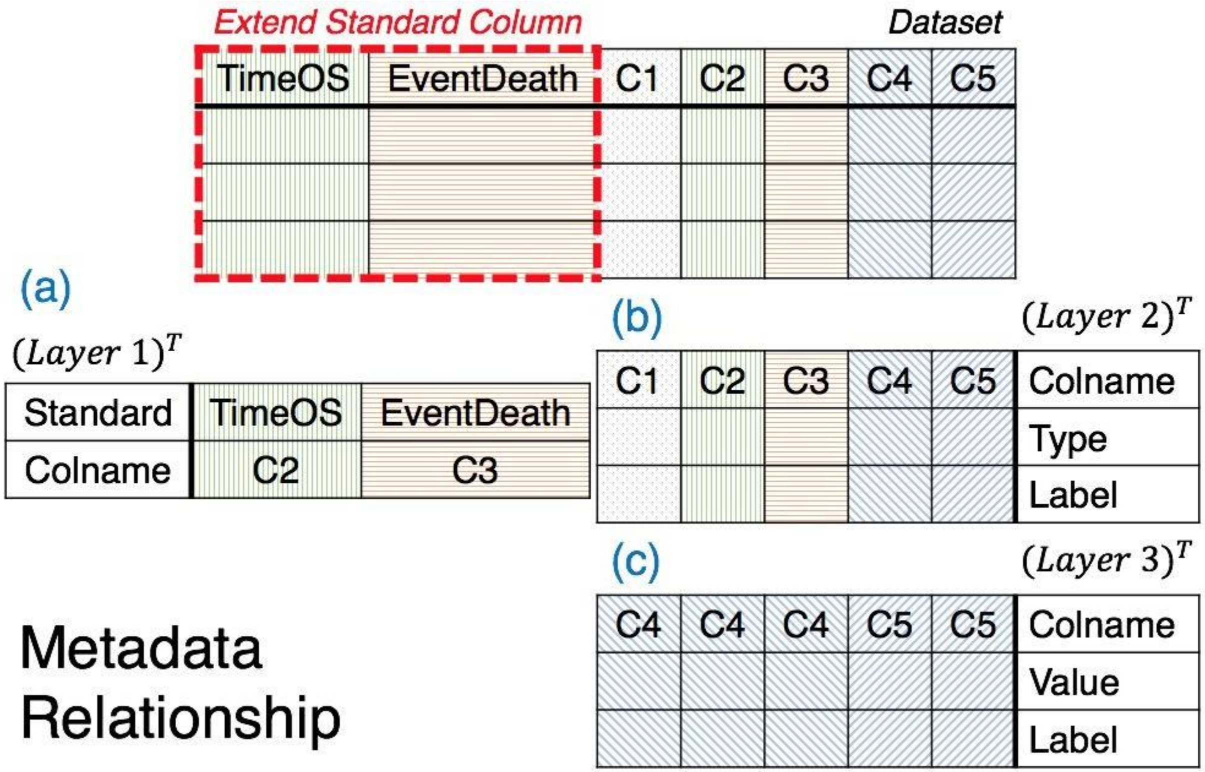

Doing biostatistic by coding

```
library(survival)

mydata<- read.csv("H:/17_hypm/hypmdd761728.csv")
attach(mydata)

# Define variables
time <- fd
event <- mdd
X <- cbind(hyp, cci)
group <- sex

# Descriptive statistics
summary(time)
summary(event)
summary(X)
summary(group)

# Kaplan-Meier non-parametric analysis
kmsurvival <- survfit(Surv(time,event) ~ 1)
summary(kmsurvival)
plot(kmsurvival, xlab="Time", ylab="Survival Probability")

# Kaplan-Meier non-parametric analysis by group
kmsurvival1 <- survfit(Surv(time, event) ~ group)
summary(kmsurvival1)
plot(kmsurvival1, lty = 2:3, col=c("red","blue"), xlab="Time", ylab="Survival Probability")
legend(20, 0.8, c("ui==0", "ui==1"), lty = 2:3, col=c("red","blue"))

# Nelson-Aalen non-parametric analysis
nasurvival <- survfit(coxph(Surv(time,event)~1), type="aalen")
summary(nasurvival)
plot(nasurvival, xlab="Time", ylab="Survival Probability")

# Cox proportional hazard model - coefficients and hazard rates
coxph <- coxph(Surv(time,event) ~ X, method="breslow")
summary(coxph)

exponential <- survreg(Surv(time,event) ~ X, dist="exponential")
summary(exponential)

weibull <- survreg(Surv(time,event) ~ X, dist="weibull")
summary(weibull)

loglogistic <- survreg(Surv(time,event) ~ X, dist="loglogistic")
summary(loglogistic)
```

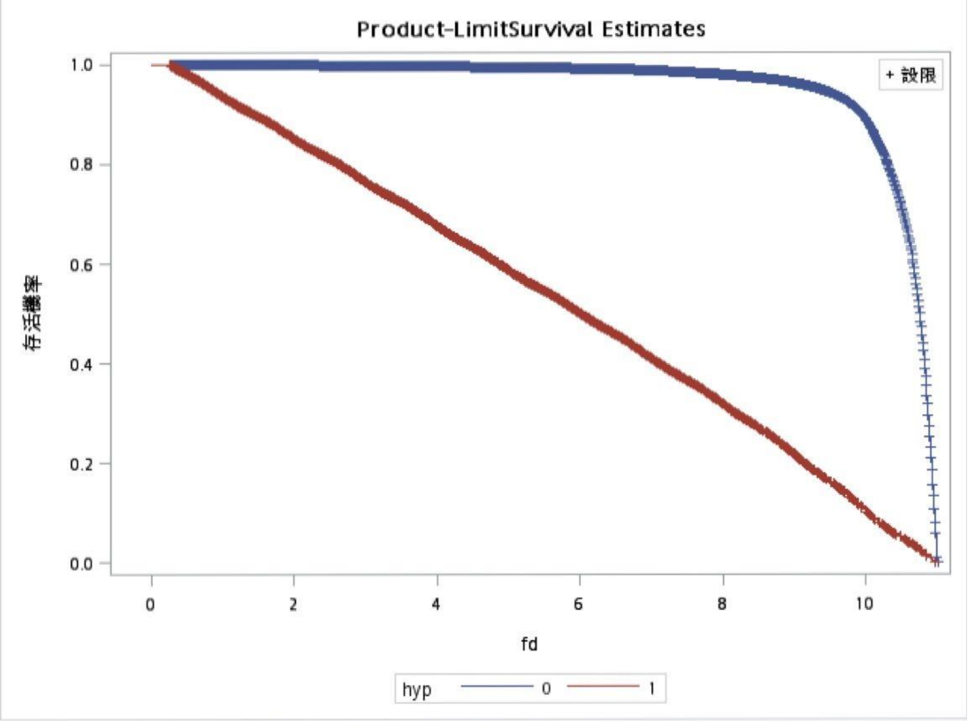

Responsive

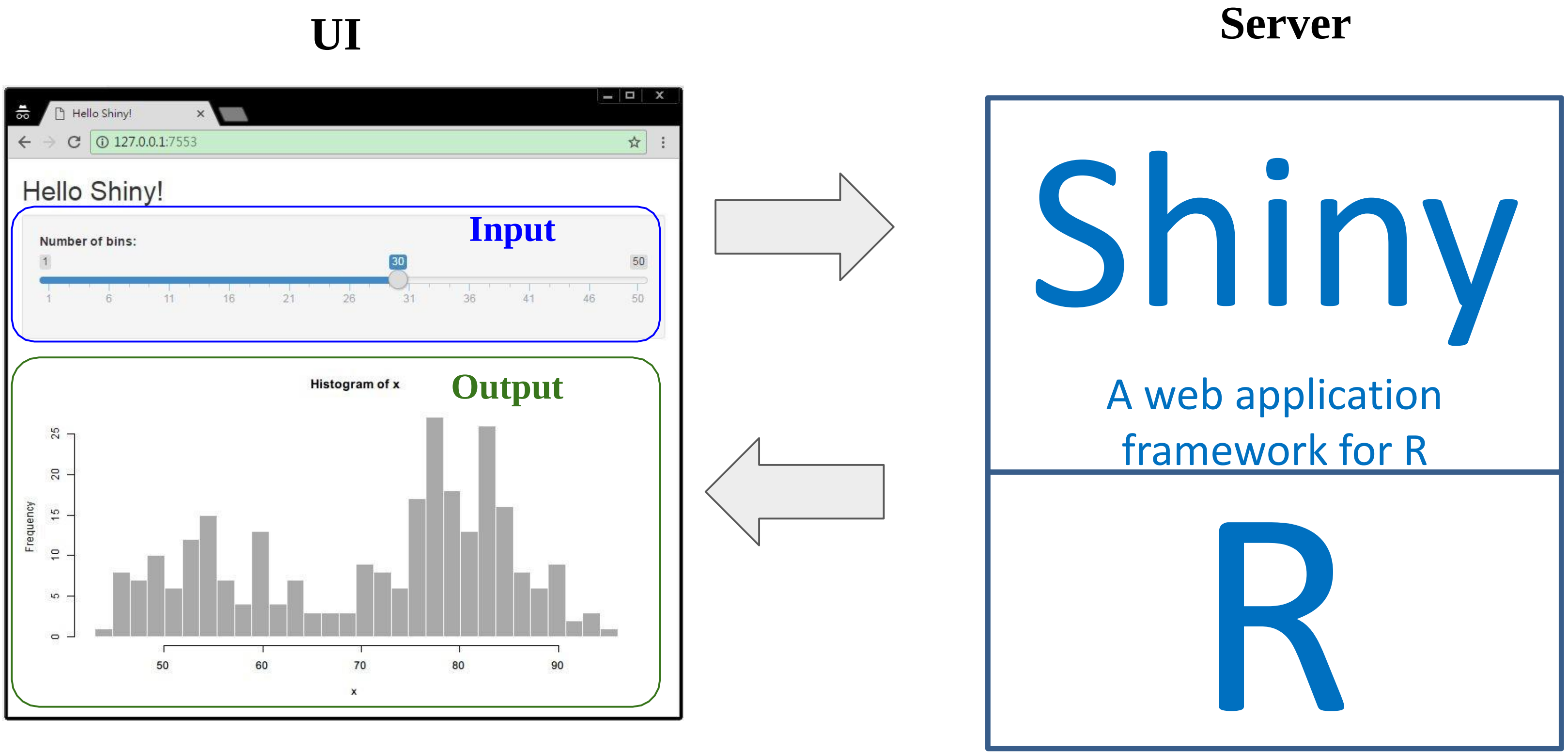
UI
Server
Hello Shiny!
127.0.0.1:7553
Hello Shiny!
Input
Number of bins:
Output
Histogram of x
Frequency
x
Shiny
A web application framework for R
R

Workflow

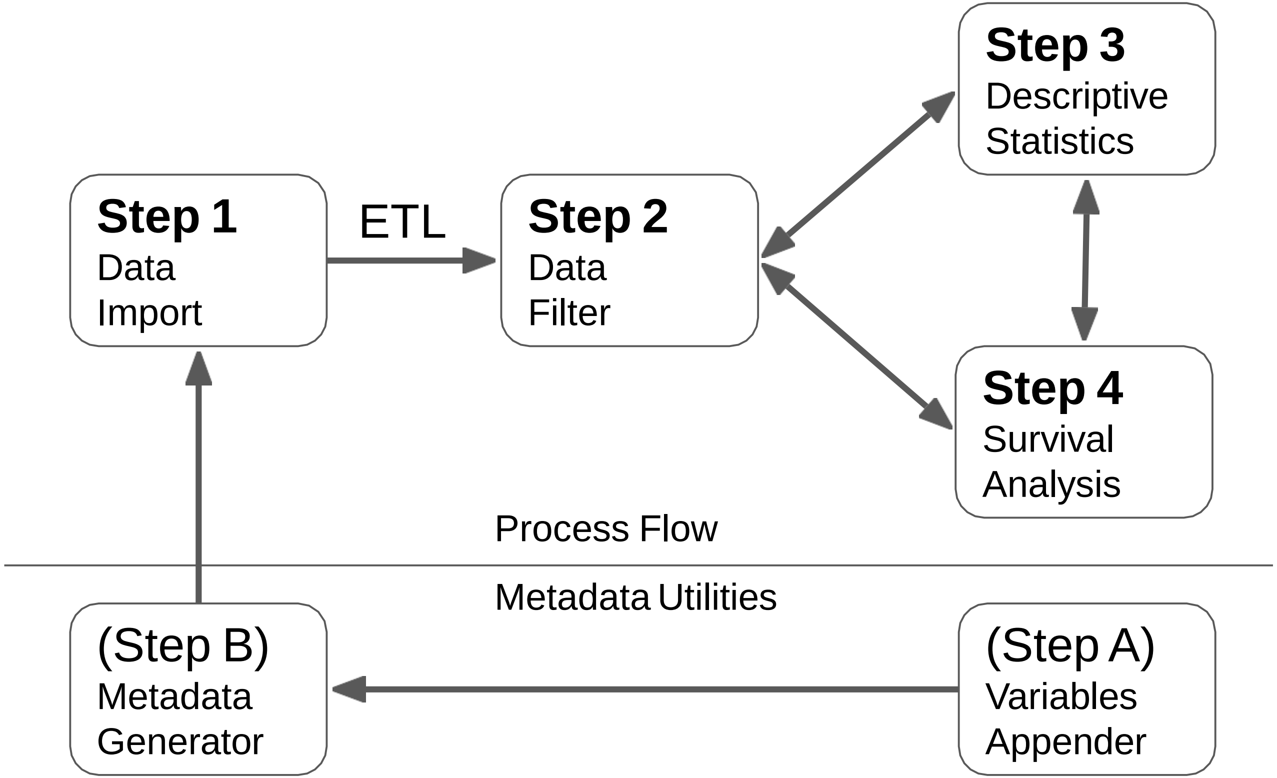

Step 1
Data
Import
ETL
Step 2
Data
Filter
Step 3
Descriptive
Statistics
Step 4
Survival
Analysis
Process Flow
Metadata Utilities
(Step B)
Metadata
Generator
(Step A)
Variables
Appender

Process Flow

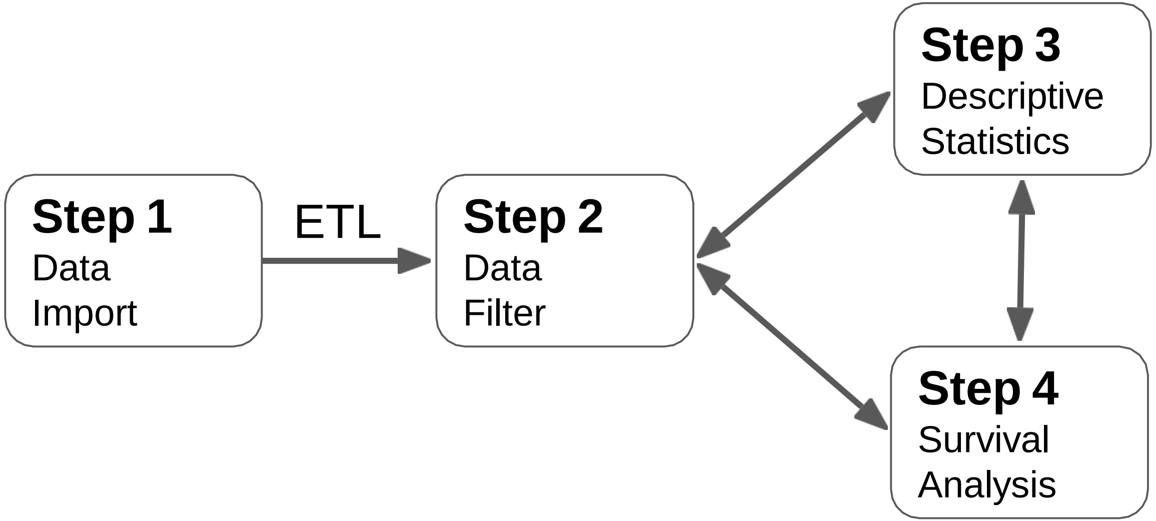

Process Flow

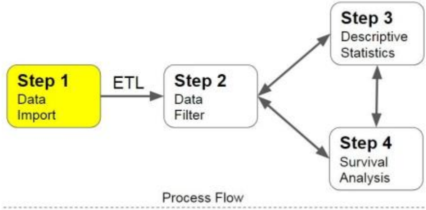

Step 1 - Data Import

Demo

Dataset

Browse... Dataset.csv

Upload complete

Metadata

Layer 1: Standard Column

Browse... Layer1.csv

Upload complete

Layer 2: Variables Manager

Browse... Layer2.csv

Upload complete

Layer 3: Categorical Reference

Browse... Layer3.csv

Upload complete

Import

Example Data

Dataset | Layer 1 | Layer 2 | Layer 3

This is your own dataset URL:

http://140.112.30.202:3838?id=00701ca6f0624b839f61881c40bfecd3

Dataset

Show 5 entries

Search:

id	study	rx	sex	age	obstruct	perfor	adhere	nodes	status	differ	extent	surg	node4	time	etype
1	1	Lev+5FU	1	43	0	0	0	5	1	2	3	0	1	1521	2
1	1	Lev+5FU	1	43	0	0	0	5	1	2	3	0	1	968	1
2	1	Lev+5FU	1	63	0	0	0	1	0	2	3	0	0	3087	2
2	1	Lev+5FU	1	63	0	0	0	1	0	2	3	0	0	3087	1
3	1	Obs	0	71	0	0	1	7	1	2	2	0	1	963	2
id	study	rx	sex	age	obstruct	perfor	adhere	nodes	status	differ	extent	surg	node4	time	etype

Showing 1 to 5 of 1,858 entries

First Previous Next Last

Layer 1: Standard Column

Standard	Colname	Description
TimeOS	time	Overall Survival Time
EventDeath	status	Flag for Death or alive (censor) (Death = 1)

Showing 1 to 2 of 2 entries

Layer 2: Variables Manager

Colname	Type	Label
id		id
study		study
rx	Nominal	treatment
sex	Nominal	sex
age	Interval	age

Showing 1 to 16 of 16 entries

Layer 3: Categorical Reference

Colname	Value	Label
rx	Obs	Observation
rx	Lev	Levamisole
rx	Lev+5FU	Levamisole+5-FU
sex	1	male
sex	0	female

Showing 1 to 28 of 28 entries

Step 2 - Data Filter

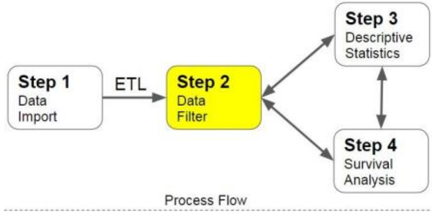

Filter Option

treatment
☐ all options ☑ Observation ☑ Levamisole ☑ Levamisole+5-FU

sex
☐ all options ☑ male ☑ female

obstruction
☐ all options ☑ yes ☑ no

perforation
☐ all options ☑ yes ☑ no

adherence
☐ all options ☑ yes ☑ no

differentiation
☑ all options ☑ well ☑ moderate ☑ poor

extent
☐ all options ☑ submucosa ☑ muscle ☑ serosa ☑ contiguous

surgery
☐ all options ☑ long ☑ short

node > 4
☐ all options ☑ yes ☑ no

event type
☐ all options ☑ recurrence ☐ death

Filter

Filter History

Expression	Before	Difference	After
treatment	1858	0	1858
sex	1858	0	1858
obstruction	1858	0	1858
perforation	1858	0	1858
adherence	1858	0	1858
extent	1858	0	1858
surgery	1858	0	1858
node > 4	1858	0	1858
event type	1858	929	929

Standardized Dataset

Show 10 entries
Search:

TimeOS	EventDeath	id	study	rx	sex	age	obstruct	perfor	adhere	nodes
968	Death	1	1	Levamisole+5-FU	male	43	no	no	no	5
3087	Censored	2	1	Levamisole+5-FU	male	63	no	no	no	1
542	Death	3	1	Observation	female	71	no	no	yes	7
245	Death	4	1	Levamisole+5-FU	female	66	yes	no	no	6
523	Death	5	1	Observation	male	69	no	no	no	22
904	Death	6	1	Levamisole+5-FU	female	57	no	no	no	9
229	Death	7	1	Levamisole	male	77	no	no	no	5
3192	Censored	8	1	Observation	male	54	no	no	no	1
3173	Censored	9	1	Levamisole	male	46	no	no	yes	2
3308	Censored	10	1	Levamisole+5-FU	female	68	no	no	no	1
TimeOS	EventDeath	id	study	rx	sex	age	obstruct	perfor	adhere	nodes

Showing 1 to 10 of 929 entries

First Previous Next Last

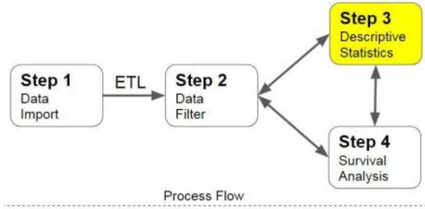

Step 3 - Demography

Demography

Stratification

EventDeath

Continuous

Show 5 entries   Search: *

Variable	Death (n = 468)	Censored (n = 461)	P value
no. of lymph nodes	3(0-33)	2(1-16)	< 0.001 *
duration	386(8-2695)	2290(23-3329)	< 0.001 *
Variable	Death (n = 468)	Censored (n = 461	P value

Showing 1 to 2 of 2 entries (filtered from 3 total entri First Previous Next Last

Categorical

Show 5 entries   Search:

Variable	Death (n = 468)	Censored (n = 461)	P value
sex			0.57
sex - male	239 (51.07%)	245 (53.15%)	
sex - female	229 (48.93%)	216 (46.85%)	
**sex**	Death (n = 468)	Censored (n = 46	P value

Showing 1 to 3 of 3 entries (filtered from 33 total ent First Previous Next Last

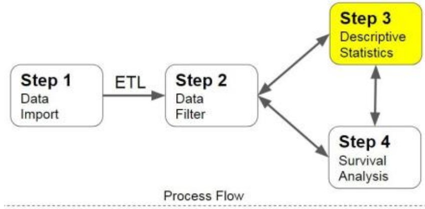

Step 3 - Pivot Table

Pivot Table

Show type

- [x] No. of patient
- [x] % of patient

Row label

sex

Sum percent by

Row | Column | Overall

Column label

EventDeath

	Death	Censored	Sum
male	239 (51.1%)	245 (53.1%)	484 (52.1%)
female	229 (48.9%)	216 (46.9%)	445 (47.9%)
Sum	468 (100%)	461 (100%)	929 (100%)

Step 3 - Epidemiology

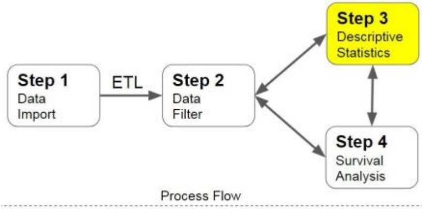

Epidemiology

Mortality

Stratification

treatment

Time unit

Day

Time unit transformer

Day to Year (/365)

Multiple Person-Time

1,000

	**Observation (n = 315)**	**Levamisole (n = 310)**	**Levamisole+5-FU (n = 304)**
Deaths (Mortality)	177 (56.19%)	172 (55.48%)	119 (39.14%)
Mortality Incidence Density per 1000 Person-Year	160.08	153.90	87.95
Sum of Person-Year	1105.73	1117.60	1353.03

Step 4 - Survival Curve

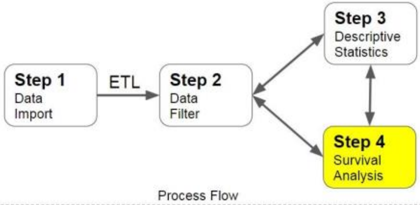

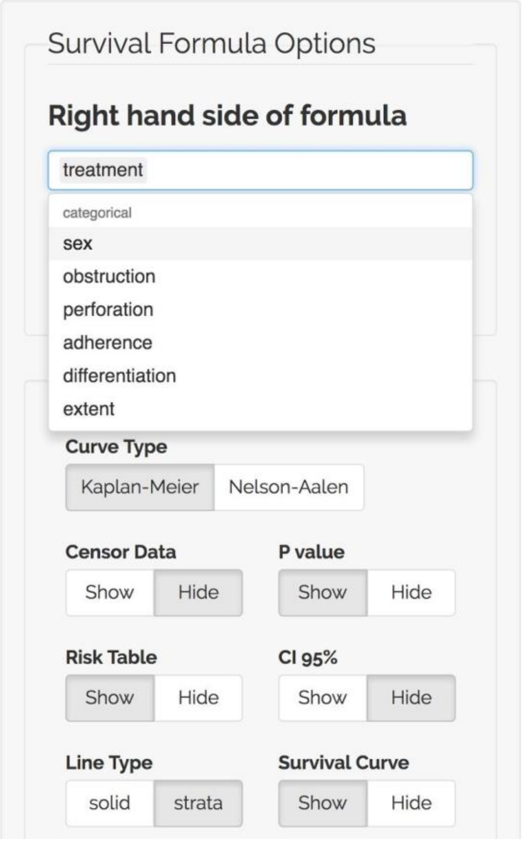

Survival Formula: Surv(TimeOS/365 *12, EventDeath) ~ rx

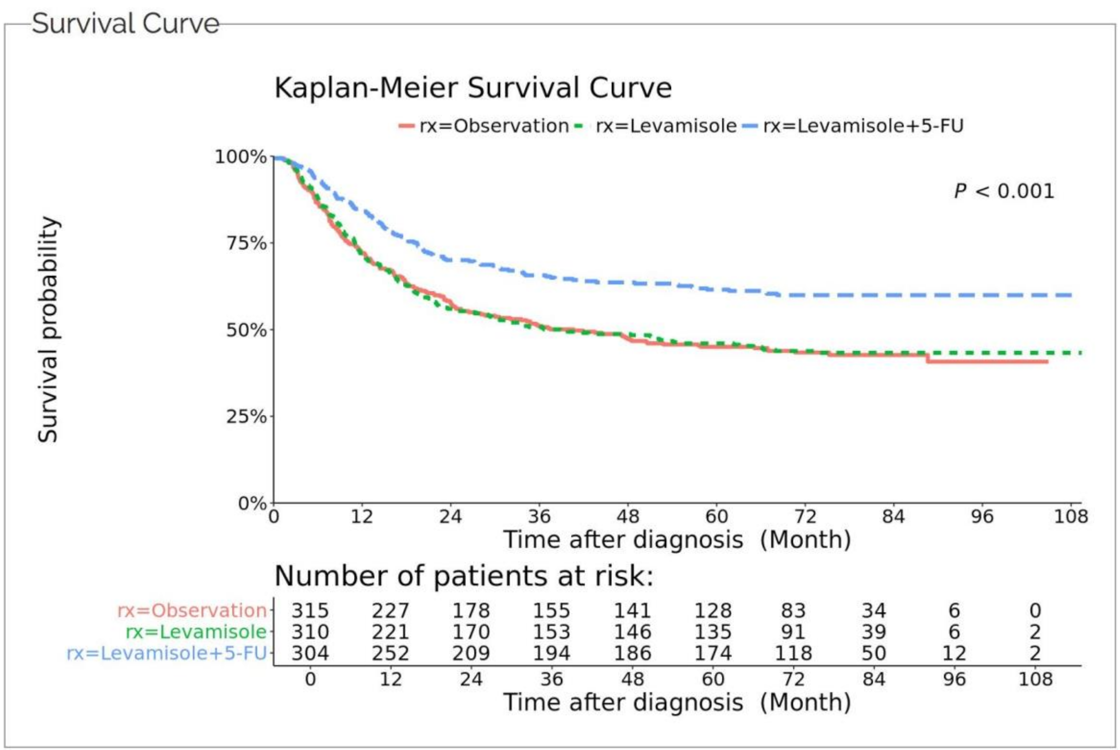

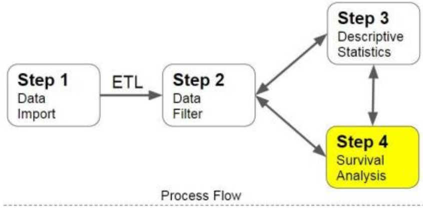

Step 4 - Survival Curve

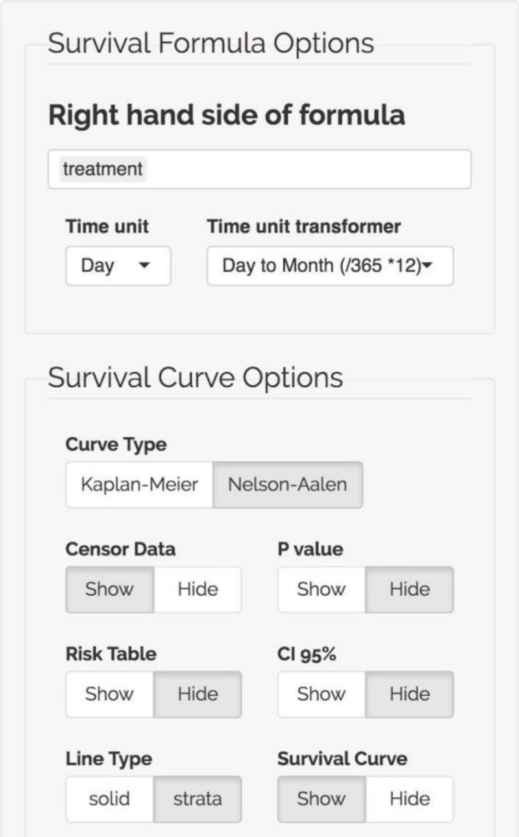

Survival Formula: Surv(TimeOS/365 *12, EventDeath) ~ rx

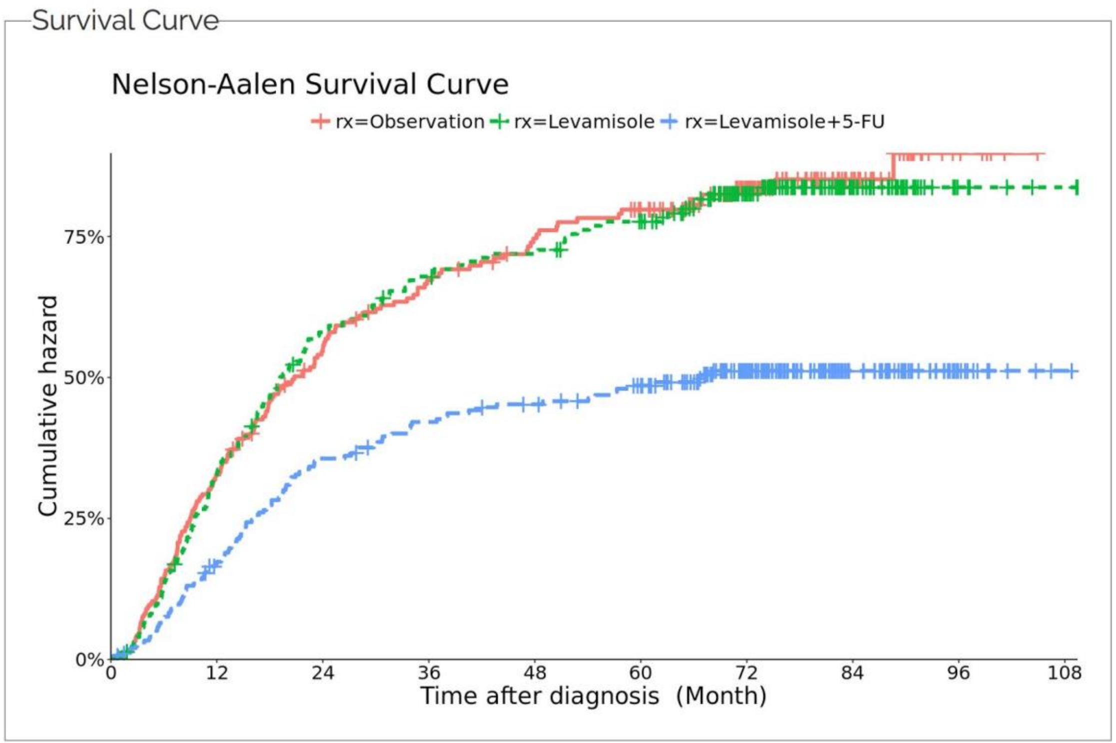

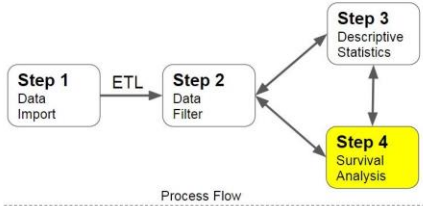

Step 4 - Cox Model

Survival Formula Options

Right hand side of formula

treatment adherence extent surgery node > 4

Time unit: Day

Time unit transformer: Day to Month (/365 *12)

Survival Curve Options

Survival Curve

Show | Hide

Cox Table Reference Category

treatment

◉ Observation ○ Levamisole ○ Levamisole+5-FU

adherence

○ yes ◉ no

extent

◉ submucosa ○ muscle ○ serosa ○ contiguous structures

surgery

○ long ◉ short

node > 4

○ yes ◉ no

Survival Formula: Surv(TimeOS/365 *12, EventDeath) ~ rx + adhere + extent + surg + node4

Cox Proportional Hazards Model

Univariate analysis

	HR	95% CI	P value	Log-rank test
treatment (Levamisole v.s. Observation)	0.98	0.8 - 1.21	0.888	23.07
treatment (Levamisole+5-FU v.s. Observation)	0.6	0.47 - 0.76	< 0.001 *	
adherence (yes v.s. no)	1.37	1.08 - 1.74	0.01 *	6.7
extent (muscle v.s. submucosa)	1.39	0.54 - 3.55	0.494	30.96
extent (serosa v.s. submucosa)	2.72	1.13 - 6.57	0.026 *	
extent (contiguous structures v.s. submucosa)	4.79	1.85 - 12.39	0.001 *	
surgery (long v.s. short)	1.29	1.06 - 1.57	0.011 *	6.43
node > 4 (yes v.s. no)	2.43	2.01 - 2.92	< 0.001 *	91.59

Multivariate analysis (Log-rank test = 147.62)

	HR	95% CI	P value
treatment (Levamisole v.s. Observation)	0.99	0.8 - 1.22	0.889
treatment (Levamisole+5-FU v.s. Observation)	0.6	0.48 - 0.76	< 0.001 *
adherence (yes v.s. no)	1.23	0.96 - 1.57	0.102
extent (muscle v.s. submucosa)	1.04	0.4 - 2.66	0.938
extent (serosa v.s. submucosa)	1.93	0.8 - 4.7	0.145
extent (contiguous structures v.s. submucosa)	3.2	1.22 - 8.36	0.018 *
surgery (long v.s. short)	1.29	1.06 - 1.58	0.011 *
node > 4 (yes v.s. no)	2.35	1.94 - 2.83	< 0.001 *

Workflow

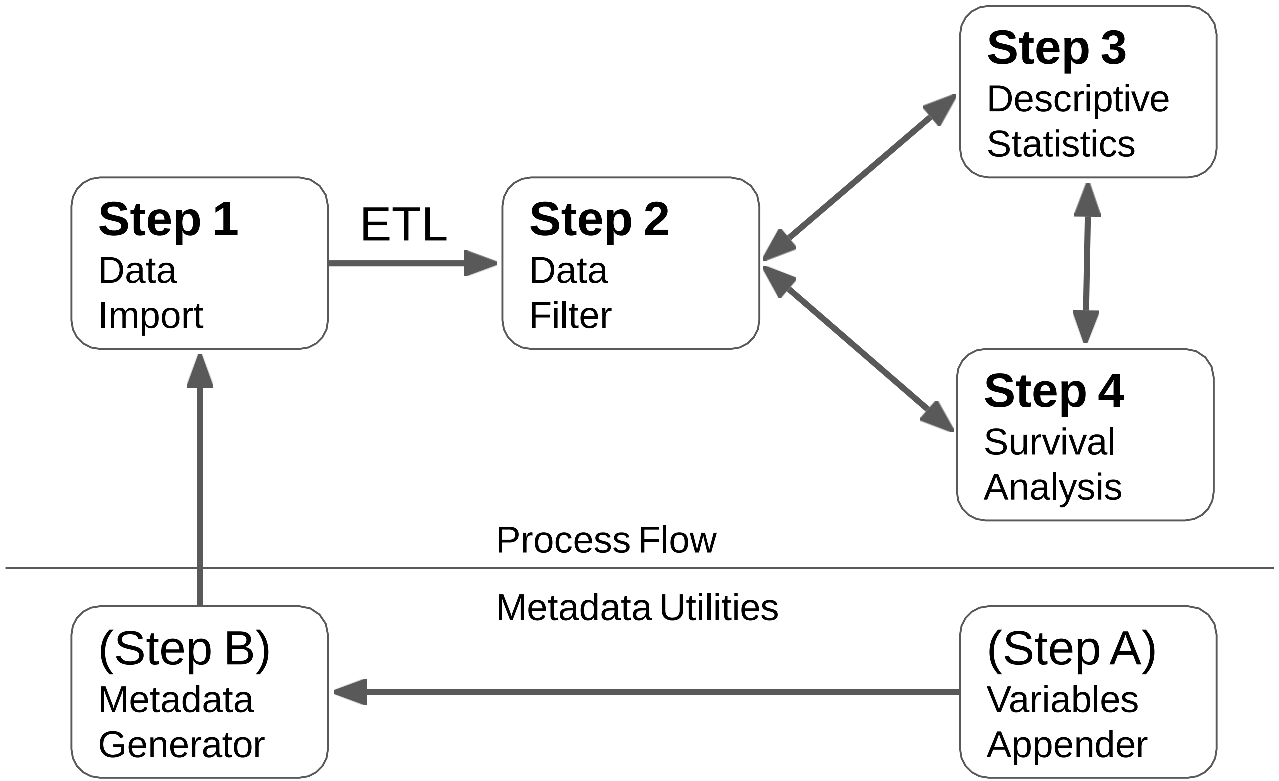
Step 1
Data
Import
ETL
Step 2
Data
Filter
Step 3
Descriptive
Statistics
Step 4
Survival
Analysis
Process Flow
Metadata Utilities
(Step B)
Metadata
Generator
(Step A)
Variables
Appender

Metadata Utilities

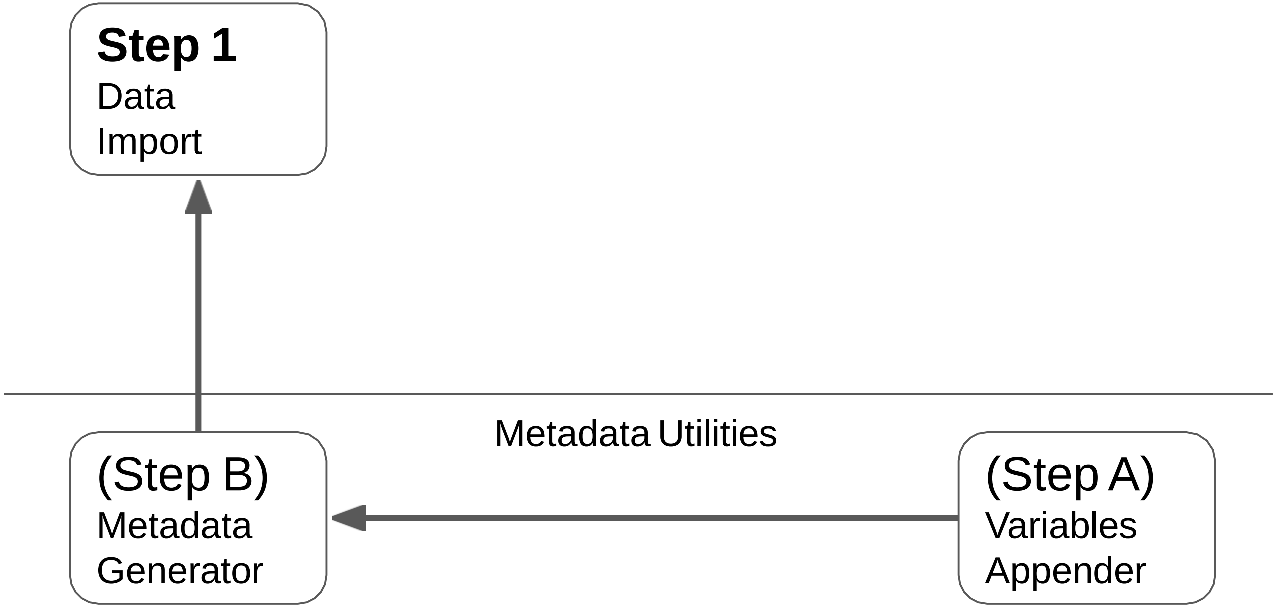

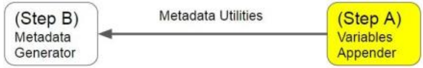

Step A - Variables Appender

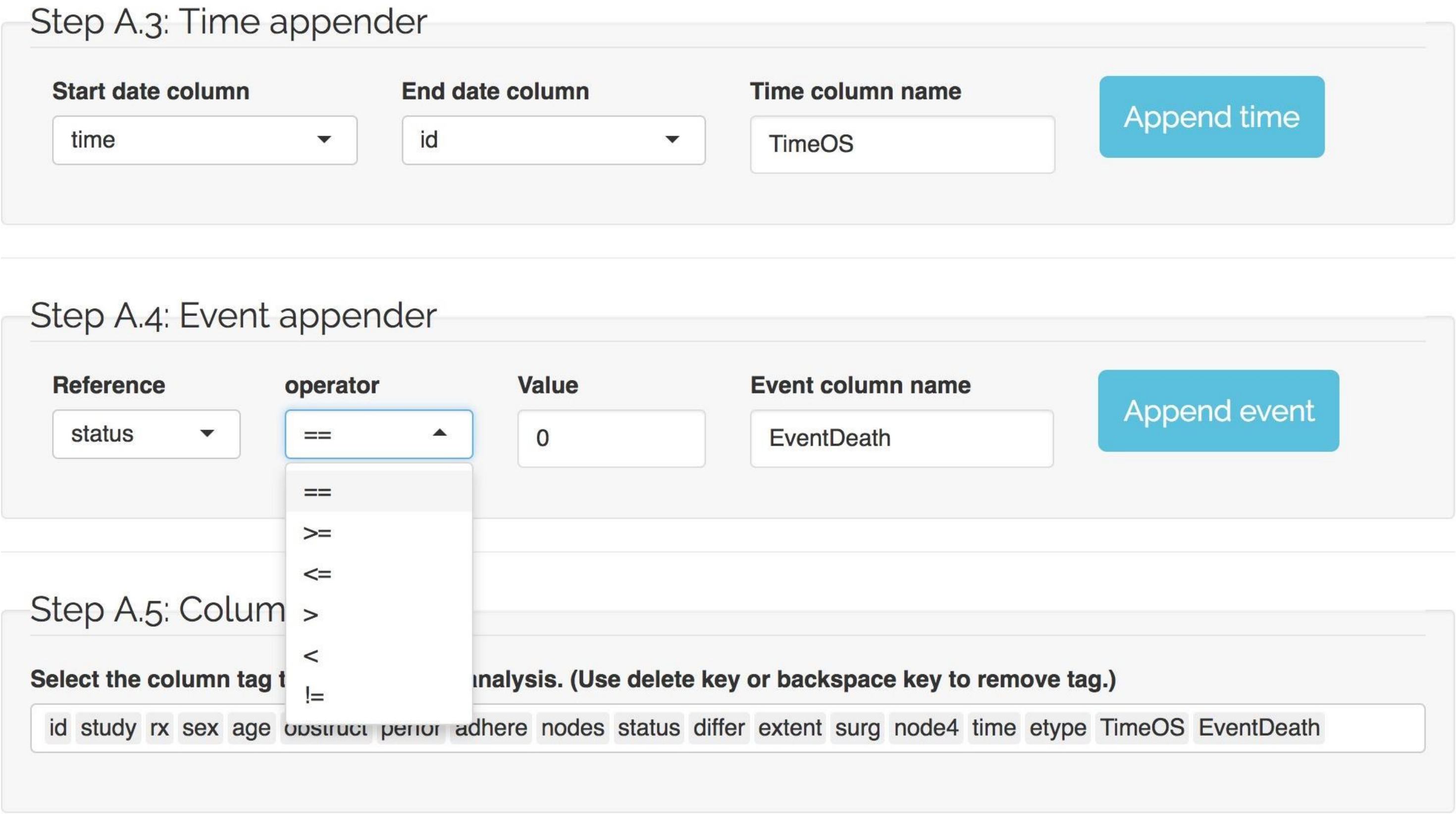

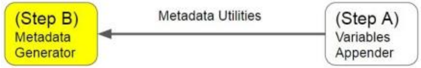

Step B - Metadata Generator

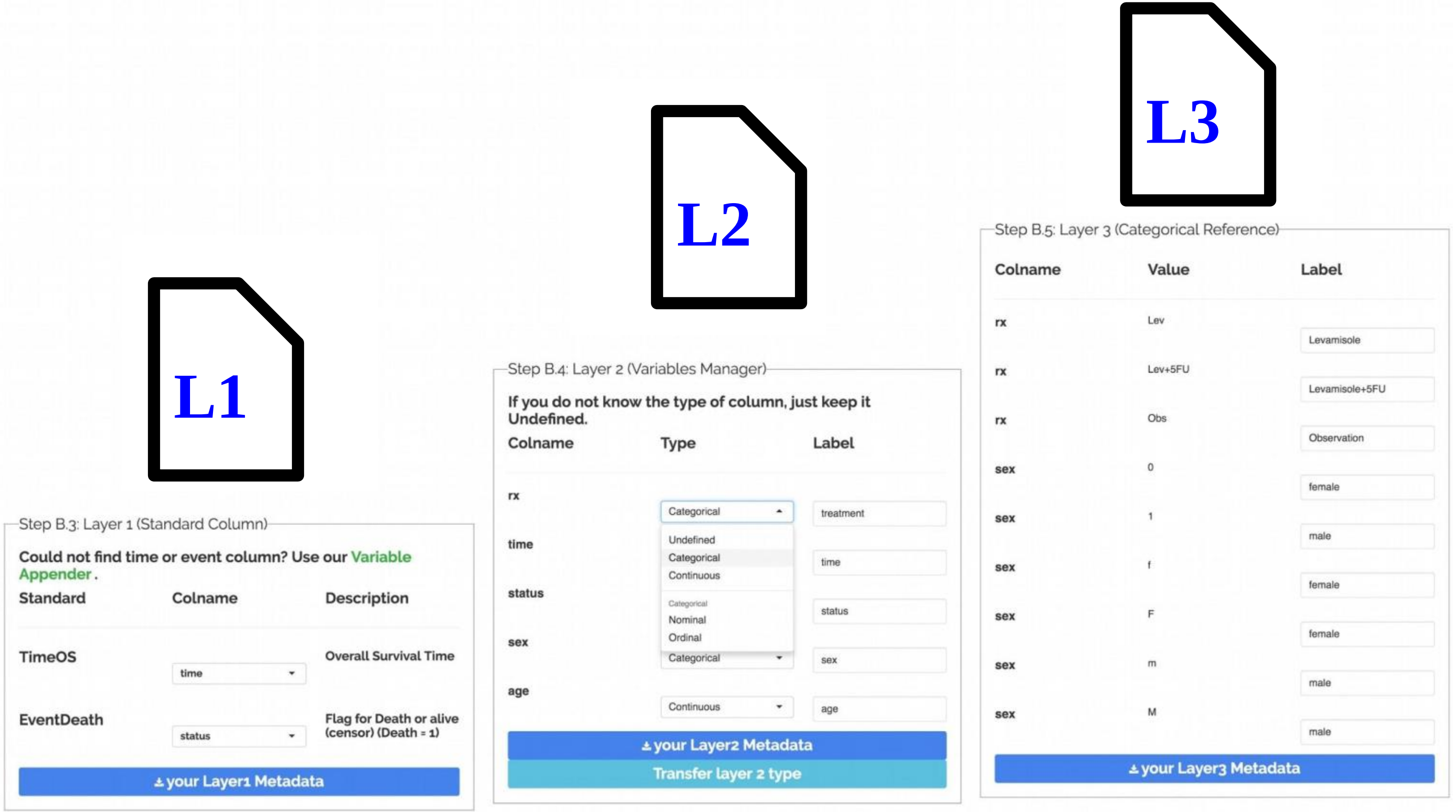

Validation

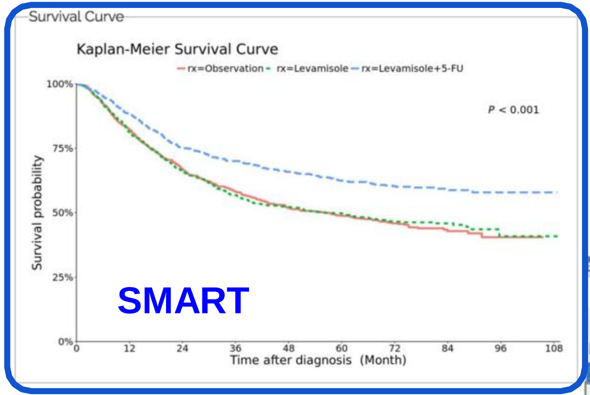
Survival Curve
Kaplan-Meier Survival Curve
rx=Observation
rx=Levamisole
rx=Levamisole+5-FU
P < 0.001
Survival probability
Time after diagnosis (Month)
SMART

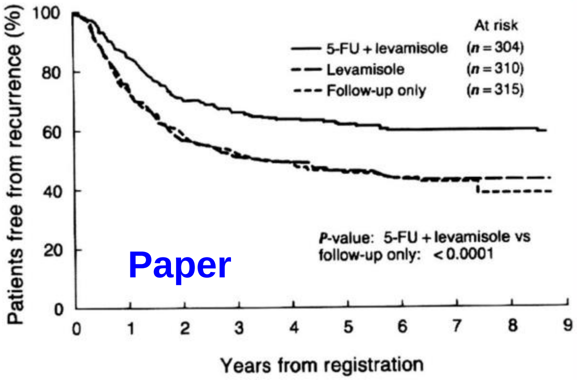
At risk
5-FU + levamisole (n = 304)
Levamisole (n = 310)
Follow-up only (n = 315)
P-value: 5-FU + levamisole vs follow-up only: < 0.0001
Patients free from recurrence (%)
Years from registration
Paper

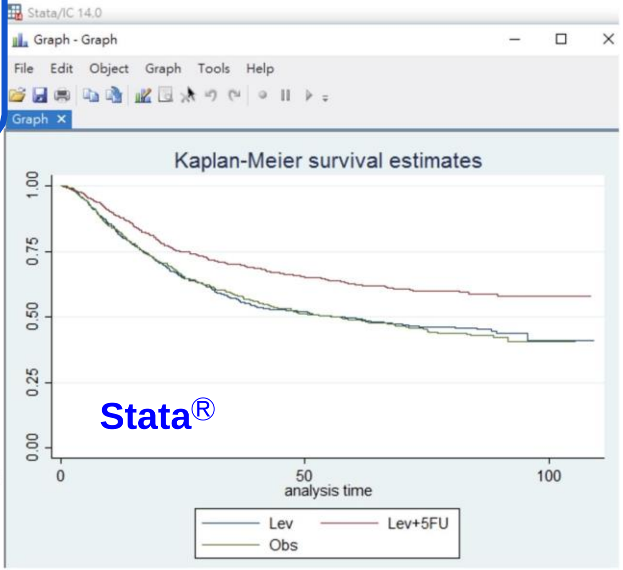
Stata/IC 14.0
Graph - Graph
File Edit Object Graph Tools Help
Graph
Kaplan-Meier survival estimates
analysis time
Lev
Lev+5FU
Obs
Stata®

Future Work

- **Internationalization:** Traditional Chinese version
- **Future Architecture:**

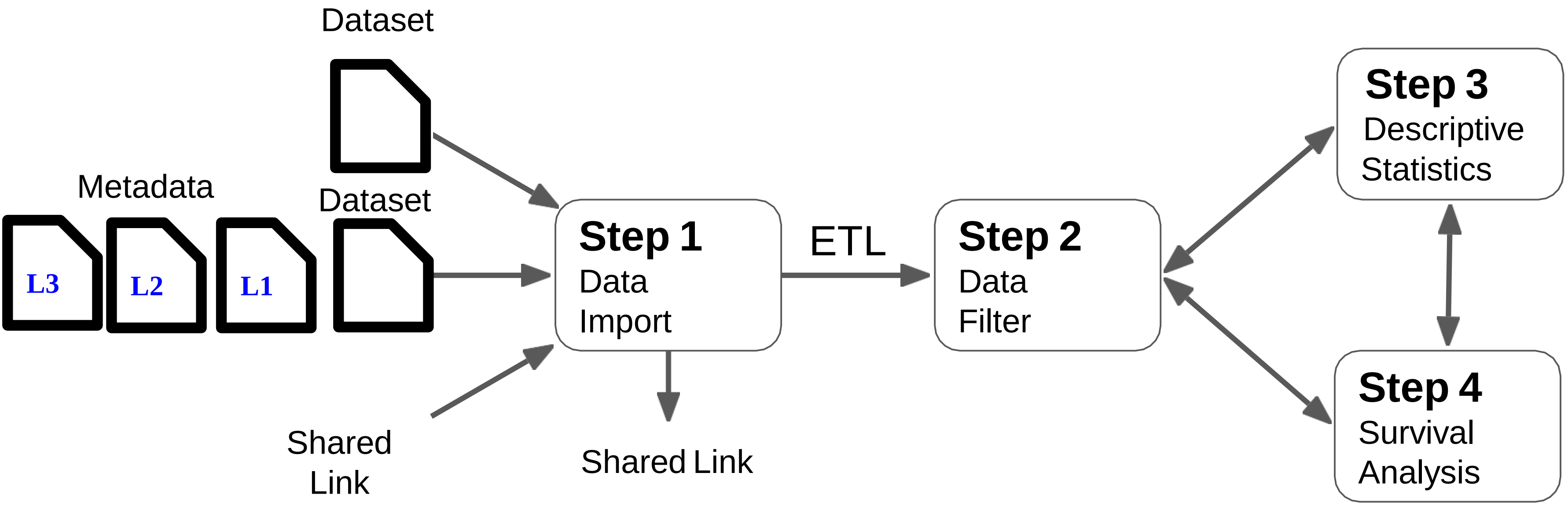

Summary

- **S**urvival **M**etadata **A**nalysis **R**esponsive **T**ool
  - **User-Friendly:** straightforward, RWD, interactive
  - **Accessibility:** free and open-source
  - **Efficiency:** automatically generate figures and tables
  - **Collaboration:** shared link and online web application
  - **Standardization:** SOP for survival analysis
  - R shiny based online web application
  - Suitable for randomized controlled trial (RCT) study.
  - Suitable for retrospective cohort study

Thank you very much

Demo website: http://140.112.30.202:3838

Contribution

- For beginners
  - Learn the standard process for survival analysis.
  - Reduce the learning threshold.
  - Don’t need to learn any statistical commands.
- For clinical researchers
  - Reduce the repetitive statistical actions.
  - Make statistical process much quicker and easier.
  - Establish an online cooperative tool for survival analysis project.

Limitation

- Suitable for randomized controlled trial (RCT) study.
- Suitable for retrospective cohort study
- Must know the basic knowledge of survival analysis.
- Must know the detail of the dataset.

Develop Environment

- **R** (v3.3.2) and **R Studio** (v1.0.136)
- R packages
  - Web: shiny_0.14.2, shinyjs_0.8, shinythemes_1.1.1
  - Data Operator: dplyr_0.5.0, nortest_1.0-4, abind_1.4-5, uuid_0.1-2
  - Survival Analysis: survival_2.40-1, survminer_0.2.4
  - Plot: ggplot2_2.2.0, ggfortify_0.3.0.0F

Hardware Environment

- Develop Computer
  - macOS Sierra @ MacBook Pro (Retina, 15-inch, Mid 2015)
  - CPU: 8 core Intel Core i7 @ 2.5 GHz
  - Memory: 16GB 1600 MHz DDR3
- Shiny Website Server
  - CentOS 7 @ IBM System x3650 M3
  - CPU: 16 core Intel(R) Xeon(R) CPU @ 2.4 GHz
  - Memory: 16G DIMM
  - Docker (v17.04.0-ce)
  - Container limit: 1G Memory
  - Loading balance with 12 container

Demostrative Dataset

- Rdatasets
  - A collection of **1072** datasets, **37** for survival analysis.
- Colon cancer dataset
  - In original paper, it is used in survival analysis.
  - **1858** records
  - **16** columns
  - **3** continuous columns
  - **10** categorical columns

id:	id
study:	1 for all patients
rx:	Treatment – Obs(ervation), Lev(amisole), Lev(amisole)+5-FU
sex:	1=male
age:	in years
obstruct:	obstruction of colon by tumour
perfor:	perforation of colon
adhere:	adherence to nearby organs
nodes:	number of lymph nodes with detectable cancer
time:	days until event or censoring
status:	censoring status
differ:	differentiation of tumour (1=well, 2=moderate, 3=poor)
extent:	Extent of local spread (1=submucosa, 2=muscle, 3=serosa, 4=contiguous structures)
surg:	time from surgery to registration (0=short, 1=long)
node4:	more than 4 positive lymph nodes
etype:	event type: 1=recurrence,2=death

https://vincentarelbundock.github.io/Rdatasets/

http://vincentarelbundock.github.io/Rdatasets/doc/survival/colon.html

Summary

- The SMART is a **free, friendly, intelligent and a highly-responsive** survival analysis web application.
- Metadata is the most critical role in the SMART.
- The Process Flow includes Data Import, Data Filter, Descriptive Statistice and Survival Analysis.
- The Metadata Utilities includes Metadata Generator and Variables Appender.
- The SMART defines a **standard operation process**.
- Establish an **online cooperative tool** for survival analysis project.

Demography Test Flow

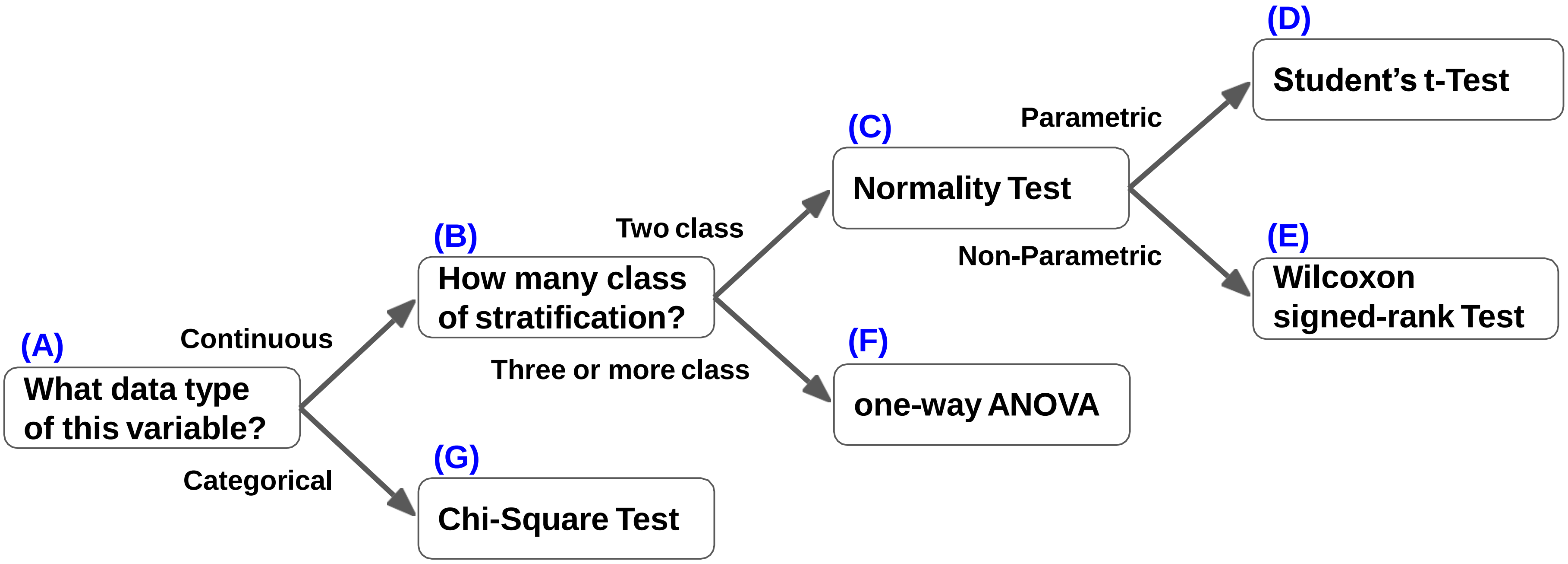

Statistics Hypothesis Test Methods

	Alternative Hypothesis (*P-value* < 0.05)	Condition
Shapiro-Wilk Test	the sample data came from a non-normal distribution population	
Anderson-Darling Test	the sample data came from a non-normal distribution population	
Student's t-Test	two sets of continuous data are significantly different	1. two sets of continuous data<br>2. normal distribution dataset
Wilcoxon signed-rank Test	two sets of continuous data are significantly different	1. two sets of continuous data<br>2. non-normal distribution dataset
one-way ANOVA	all sets of continuous data are significantly different	two or more sets of continuous data
Pearson's Chi-square Test	two or more sets of categorical data are significantly different	two or more sets of categorical data
Log-Rank Test	the specific category is significantly different from reference base category	

Mortality Incidence Density

$$Mortality = \frac{The\ count\ of\ death\ people}{The\ count\ of\ total\ sick\ people} \times 100\ (\%)$$

$$\text{Mortality Incidence Density} = \alpha * \frac{D}{\sum_{i=1}^{n}(T_i)}$$

Per Person-Time

- α is a magnification, researchers often use 1000
- The D is the count of dead people.
- The T_i is life span of the i_{th} person.

Statistics of Survival Analysis

- **Kaplan-Meier**

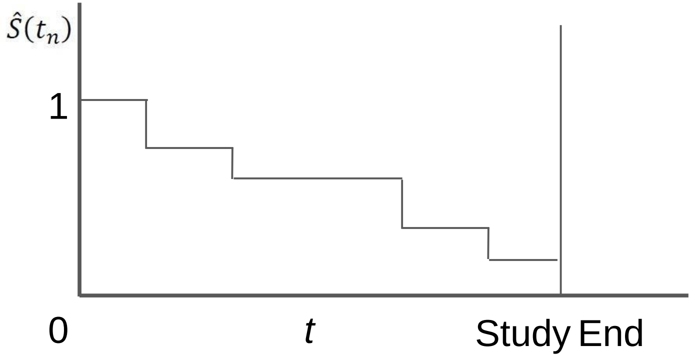

$$\hat{S}(t_n) = \prod_{i=1}^{n} \widehat{Pr}[T > t_i \mid T \geq t_i] = \hat{S}(t_{n-1}) \times \widehat{Pr}[T > t_n \mid T \geq t_n]$$

- **Nelson-Aalen**

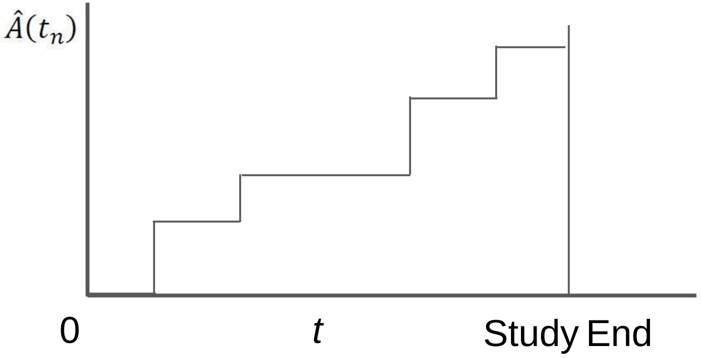

$$\hat{A}(t_n) = \sum_{i=1}^{n} \frac{d_i}{r_i}$$

d_j is the number of deaths

r_j is the number of alive

$$\mathrm{A(t)} = -\ln[S(t)]$$

- **Cox Proportional Hazards Model**

$$\log(\mathrm{HR}) = \log\left(\frac{h(t)}{h_0(t)}\right) = \beta_1 X_1 + \beta_2 X_2 + \cdots + \beta_p X_p$$

R and R Packages

- R
  - statistical programming language
  - open-source freely available software
  - RStudio: development tool
  - CRAN (Comprehensive R Archive Network)
- R Packages
  - survival package
    - survival analysis
  - shiny package
    - web application framework
    - responsive tool
  - dplyr package
    - data manipulation tool

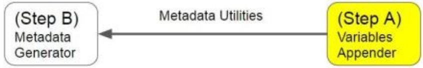

A.1 ~ A.2 - Upload & Check dataset

Step A.1: Upload raw dataset

Raw Dataset

Browse... No file selected

Import dataset

Import

Example dataset

Demo

Step A.2: Check raw dataset

Show 5 entries

Search:

id	study	rx	sex	age	obstruct	perfor	adhere	nodes	status	differ	extent
1	1	Lev+5FU	1	43	0	0	0	5	1	2	3
1	1	Lev+5FU	1	43	0	0	0	5	1	2	3
2	1	Lev+5FU	1	63	0	0	0	1	0	2	3
2	1	Lev+5FU	1	63	0	0	0	1	0	2	3
3	1	Obs	0	71	0	0	1	7	1	2	2
id	study	rx	sex	age	obstruct	perfor	adhere	nodes	status	differ	extent

Showing 1 to 5 of 1,858 entries

First Previous Next Last

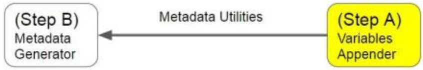

A.3 ~ A.4 - Time and Event Appender

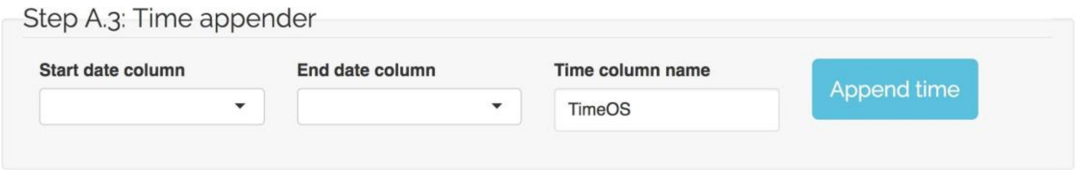

Time = End date - Start date

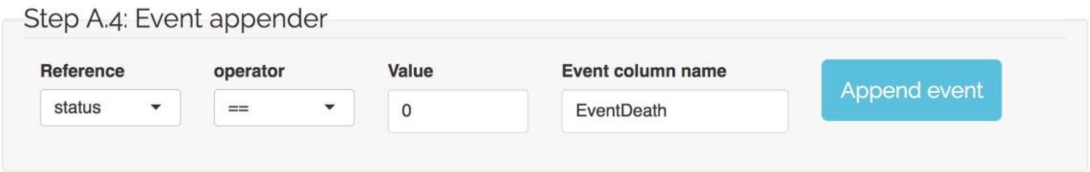

Boolean operator

1 = TRUE, 0 = FALSE

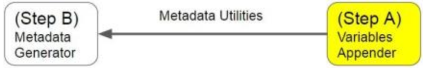

A.5 ~ A.7 - Selector, Check, Download

Step A.5: Column selector

Select the column tag that you want to analysis. (Use delete key or backspace key to remove tag.)

id rx sex age status time EventDeath

Step A.6: Processed dataset

Show 5 entries

Search:

id	rx	sex	age	status	time	EventDeath
1	Lev+5FU	1	43	1	1521	0
1	Lev+5FU	1	43	1	968	0
2	Lev+5FU	1	63	0	3087	1
2	Lev+5FU	1	63	0	3087	1
3	Obs	0	71	1	963	0
id	rx	sex	age	status	time	EventDeath

Showing 1 to 5 of 1,858 entries

First Previous Next Last

Step A.7: Download Processed Dataset

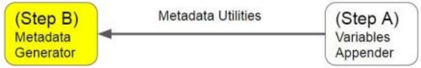

B.1 ~ B.2 - Upload & Check dataset

Step B.1: Upload dataset

Dataset name

Dataset

Browse... No file select

Import dataset

Import

Example dataset

Demo

Step B.2: Check dataset

Show 5 entries

Search:

id	study	rx	sex	age	obstruct	perfor	adhere	nodes	status	differ	extent
1	1	Lev+5FU	1	43	0	0	0	5	1	2	3
1	1	Lev+5FU	1	43	0	0	0	5	1	2	3
2	1	Lev+5FU	1	63	0	0	0	1	0	2	3
2	1	Lev+5FU	1	63	0	0	0	1	0	2	3
3	1	Obs	0	71	0	0	1	7	1	2	2
id	study	rx	sex	age	obstruct	perfor	adhere	nodes	status	differ	extent

Showing 1 to 5 of 1,858 entries

First Previous Next Last

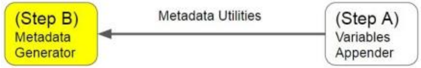

B.3 - Layer 1 Metadata (filled)

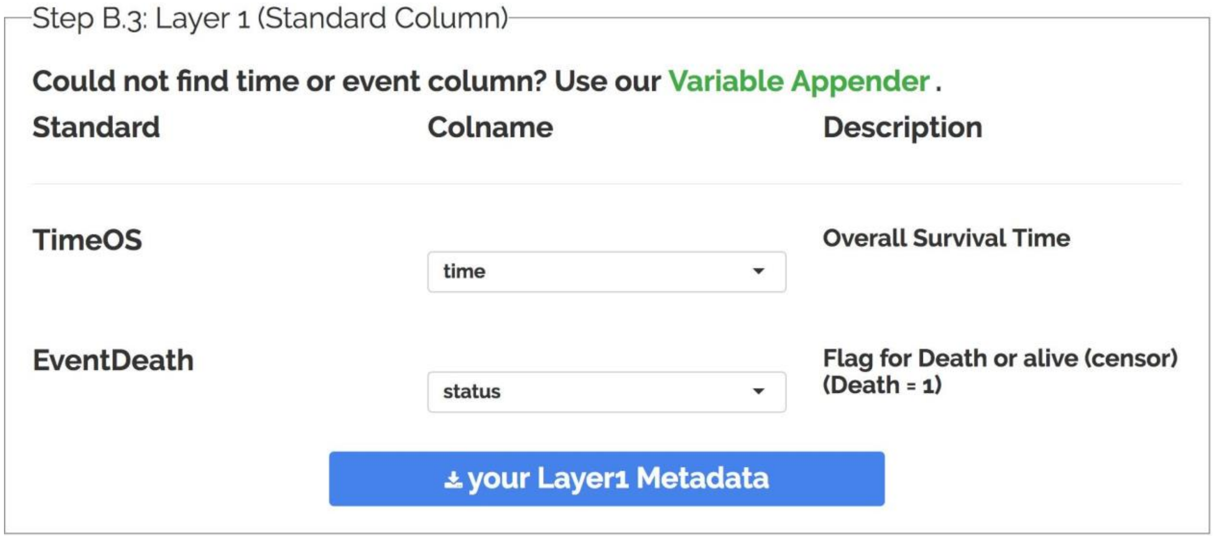

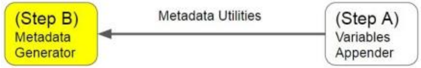

B.4 - Layer 2 Metadata (default)

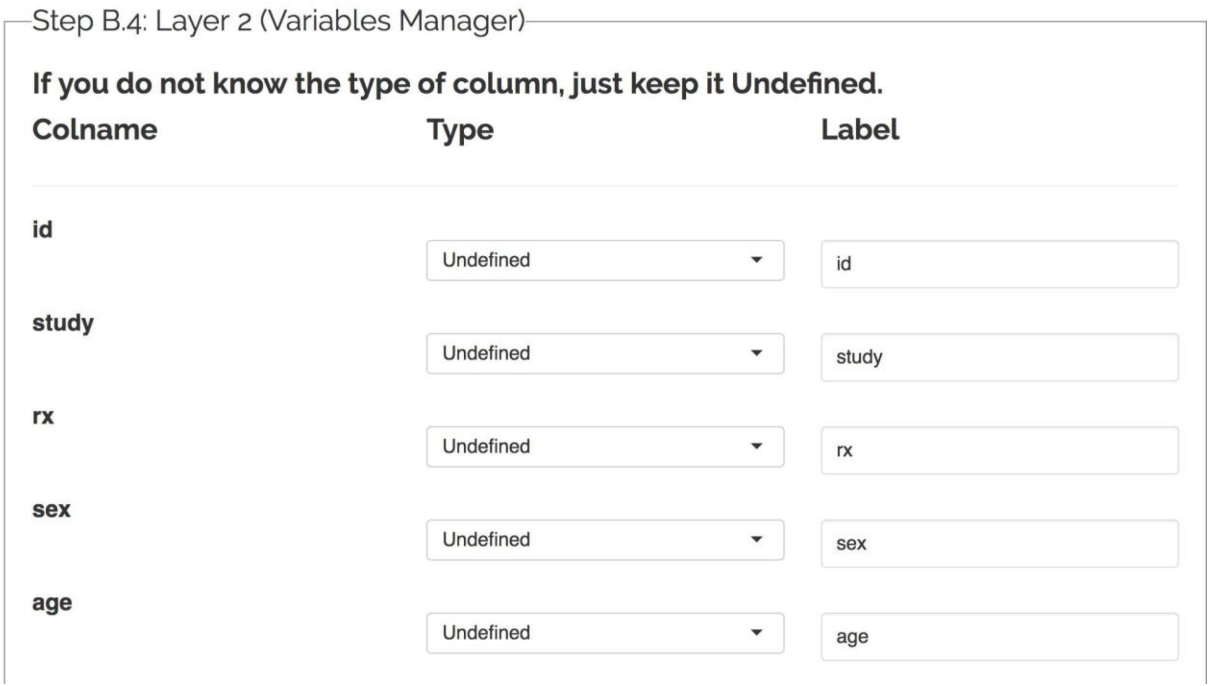

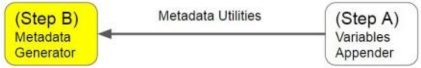

B.4 - Layer 2 Metadata (filled)

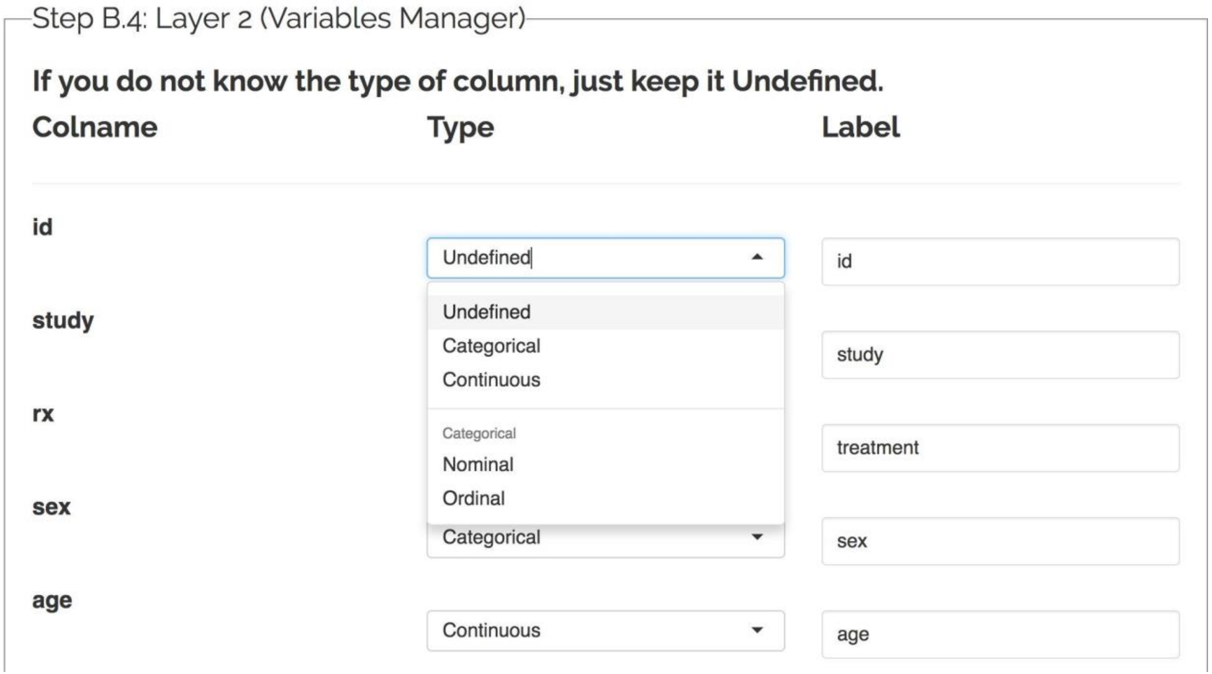

B.5 - Layer 3 Metadata

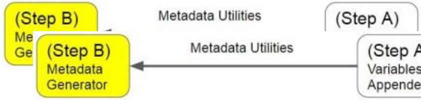

your Layer2 Metadata

Transfer layer 2 type

Step B.5: Layer 3 (Categorical Reference)

Colname	Value	Label
rx	Obs	Obs
rx	Lev	Lev
rx	Lev+5FU	Lev+5FU
sex	1	yes
sex	0	no

your Layer3 Metadata

Step B.5: Layer 3 (Categorical Reference)

Colname	Value	Label
rx	Obs	Observation
rx	Lev	Levamisole
rx	Lev+5FU	Levamisole+5-FU
sex	1	male
sex	0	female

your Layer3 Metadata

SPSS Price

Subscription	Perpetual license

Base	Standard	Professional	Premium
Single-user, desktop application for Windows and Macs. Includes 12 months of technical support.	Single-user, desktop application for Windows and Macs. Includes 12 months of technical support.	Single-user, desktop application for Windows and Macs. Includes 12 months of technical support.	Single-user, desktop application for Windows and Macs. Includes 12 months of technical support.
Starting at $ 1,210 00 USD* per year	Starting at $ 2,690 00 USD* per year	Starting at $ 5,400 00 USD* per year	Starting at $ 8,050 00 USD* per year
Purchase now	Purchase now	Purchase now	Purchase now

New purchases

Government/nonprofit single-user

Stata/IC	Stata/SE	Stata/MP 2-core	Stata/MP 4-core	Stata/MP + cores
For mid-sized datasets.	For large datasets.	Fast & for the largest datasets.	Faster.	Even faster.
Perpetual ▼	Perpetual ▼	Perpetual ▼	Perpetual ▼	Select cores ▼
**$1,195**/perpetual Buy	**$1,695**/perpetual Buy	**$1,995**/perpetual Buy	**$2,295**/perpetual Buy	

**Product features**	**Stata/IC**	**Stata/SE**	**Stata/MP**		
Maximum number of variables	2,048	32,767	120,000		
Maximum number of observations	2.14 billion	2.14 billion	Up to 20 billion		
Maximum number of independent variables	798	10,998	10,998		
**Multicore support**	**1-core**	**1-core**	**2-core**	**4-core**	**4+**
Time to run logistic regression with 5 million obs and 10 covariates	10.0 sec	10.0 sec	5.0 sec	2.6 sec	Even faster

shinyapps.io by RStudio

Home Features Pricing Support Log In

FREE	STARTER	BASIC	STANDARD	PROFESSIONAL
$ 0 /month	$ 9 /month (or $100/year)	$ 39 /month (or $440/year)	$ 99 /month (or $1,100/year)	$ 299 /month (or $3,300/year)
New to Shiny? Deploy your applications for FREE.	More applications. More active hours!	Take your users to the next level!	Password protection? Authenticate your users!	Professional has it all! Personalize your domains.
**5** Applications	**25** Applications	**Unlimited** Applications	**Unlimited** Applications	**Unlimited** Applications
**25** Active Hours	**100** Active Hours	**500** Active Hours	**2,000** Active Hours	**10,000** Active Hours
Community Support	Premium Support	Performance Boost	Authentication	Authentication
RStudio Branding		Premium Support	Performance Boost	Account Sharing
			Premium Support	Performance Boost
				Custom Domains
				Premium Support
Sign Up Now	Sign Up Now	Sign Up Now	Sign Up Now	Sign Up Now

RStudio Shiny Server Pro Pricing

Publish Shiny applications on RStudio Shiny Server Pro.

	RStudio Shiny Server Pro<br>$9,995 per year<br>BUY NOW
Concurrent Users	20
Upgrade Path	Add Concurrent User packs of 20 for $4,995 and 150 for $14,995
Commercial License (not AGPL)	●
RStudio Support	●
Deploy Shiny apps and interactive documents to the internet	●
Controlled access via SSL and LDAP, Active Directory, Google OAuth, PAM, proxied authentication, or passwords	●
Tune & Scale applications across multiple processes	●
Metrics & Session Management	●
Pricing	$9,995/yr
	BUY NOW

HAProxy - Loading Balance

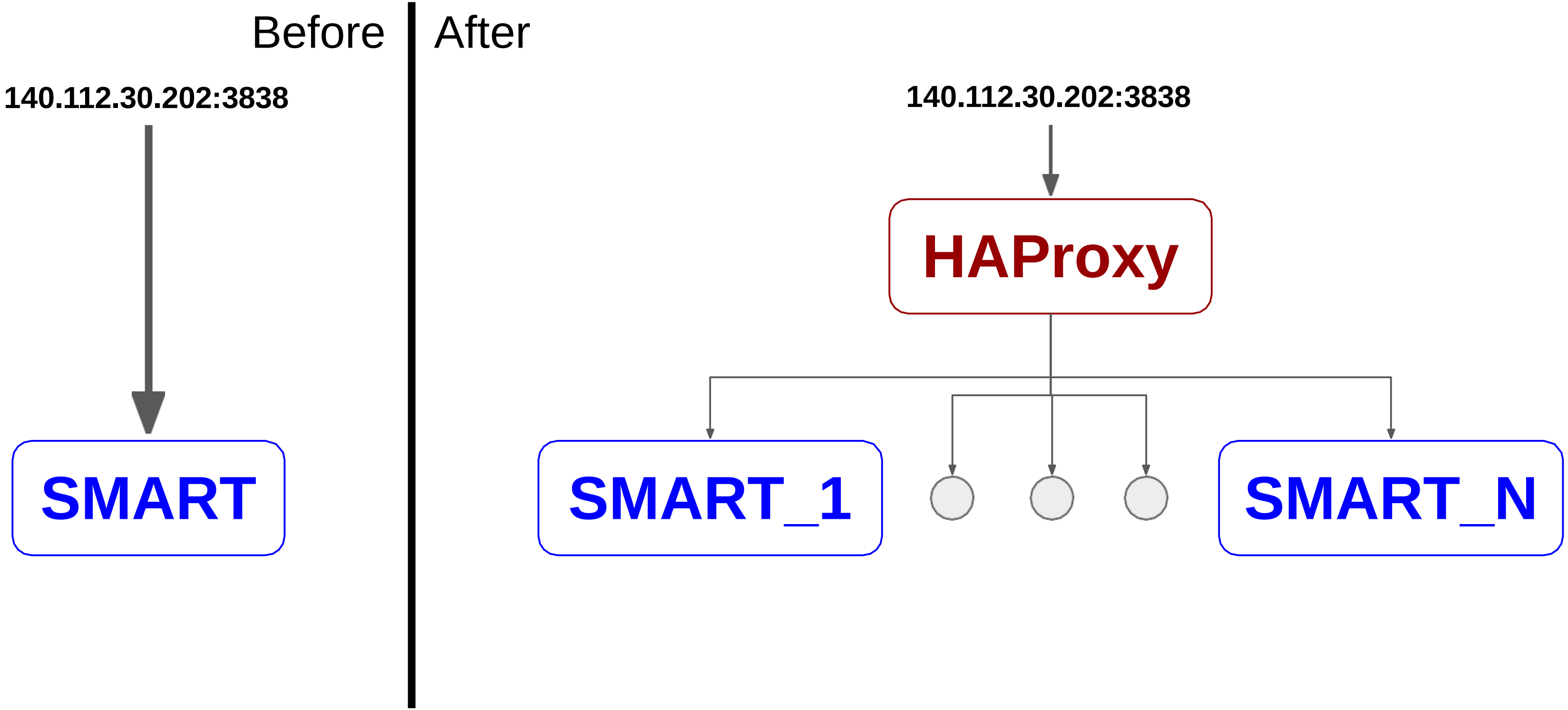

Supplement: Supplementary file 1 — SMART Supplementary [file 41598_2018_31290_MOESM1_ESM.pdf]
